# Sense and antisense RNA products of the *uxuR* gene can affect motility and chemotaxis acting independent of the UxuR protein

**DOI:** 10.3389/fmolb.2023.1121376

**Published:** 2023-02-17

**Authors:** Maria N. Tutukina, Artemiy I. Dakhnovets, Anna D. Kaznadzey, Mikhail S. Gelfand, Olga N. Ozoline

**Affiliations:** ^1^ Center for Molecular and Cellular Biology, Skolkovo Institute of Science and Technology, Moscow, Russia; ^2^ Lab of Functional Genomics and Cellular Stress, Institute of Cell Biophysics RAS, FRC PRCBR, Pushchino, Russia; ^3^ RTC “Bioinformatics”, A. A. Kharkevich Institute for Information Transmission Problems RAS, Moscow, Russia; ^4^ Department of Biotechnology, M. V. Lomonosov Moscow State University, Moscow, Russia

**Keywords:** antisense transcription, bacteria, sRNA, exoRNA, UxuR, metabolic regulation, motility

## Abstract

Small non-coding and antisense RNAs are widespread in all kingdoms of life, however, the diversity of their functions in bacteria is largely unknown. Here, we study RNAs synthesised from divergent promoters located in the 3′-end of the *uxuR* gene, encoding transcription factor regulating hexuronate metabolism in *Escherichia coli.* These overlapping promoters were predicted *in silico* with rather high scores, effectively bound RNA polymerase *in vitro* and *in vivo* and were capable of initiating transcription in sense and antisense directions. The genome-wide correlation between *in silico* promoter scores and RNA polymerase binding *in vitro* and *in vivo* was higher for promoters located on the antisense strands of the genes, however, sense promoters within the *uxuR* gene were more active. Both regulatory RNAs synthesised from the divergent promoters inhibited expression of genes associated with the *E. coli* motility and chemotaxis independent of a carbon source on which bacteria had been grown. Direct effects of these RNAs were confirmed for the *fliA* gene encoding σ^28^ subunit of RNA polymerase. In addition to intracellular sRNAs, promoters located within the *uxuR* gene could initiate synthesis of transcripts found in the fraction of RNAs secreted in the extracellular medium. Their profile was also carbon-independent suggesting that intragenic *uxuR* transcripts have a specific regulatory role not directly related to the function of the protein in which gene they are encoded.

## 1 Introduction

Although non-coding transcripts have already been studied for quite a long time, their new biotypes (Markelova et al., 2021; Diallo et al., 2022) and ways of functioning (Hör et al., 2020) are still being discovered. The ability of such transcripts to form duplexes with mRNA allows them to affect almost all stages of gene expression, from transcription and translation to RNA degradation. In the last decades, dozens of candidate regulatory RNAs were revealed or predicted but in most cases their modes of action and biological role are still rather obscure (The ENCODE Project Consortium, 2007; Dornenburg et al., 2010; Chao and Vogel, 2016).

The first discovered and the best characterised regulatory RNAs in bacteria are small regulatory RNAs (sRNAs), ranging from dozens to hundreds of nucleotides in length. They can form complementary or partly complementary duplexes with mRNAs or their regulatory regions, e.g., ribosome binding sites, thus affecting translation or stability of mRNAs (Gottesman and Storz, 2011; Hör et al., 2020).

More recently, regulatory functions were described for the fragments originating from tRNAs (tRFs) and the 3′-ends of mRNAs (Lalaouna et al., 2015; Miyakoshi et al., 2015; Chao and Vogel, 2016). Very intriguing are extracellular RNAs (exoRNAs), with a potential to sense and to transmit environmental changes (Ghosal et al., 2015; Alikina et al., 2018; Markelova et al., 2021), and even to mimic eukaryotic microRNAs thus targeting host immune response (Sahr et al., 2022).

The two questions that are frequently being asked about exoRNAs are: how they are secreted and how they remain stable? It is believed that the exoRNA transport mainly proceeds *via* the extracellular membrane vesicles (EV), which protect them from degradation by nucleases (Ozoline and Jass, 2019). Since EVs represent a conserved communication mechanism, being involved in host colonization, biofilm formation, transfer of antimicrobial resistance, and modulation of the immune response, it had also been believed that EVs may play a key role in the effects of exoRNAs. However, in (Dauros-Singorenko et al., 2020) it was clearly shown that the EV-associated RNA cargo was not involved in any significant transcriptional changes or cytokine secretion by bladder cells. Thus, the question about the mechanisms of exoRNAs action still remains open.

The majority of exoRNAs are rather short, not longer than 50 bases. Many of them can base pair with similarly cut fragments synthesized from the opposite strand, forming microRNA-sized RNAs (Dauros-Singorenko et al., 2018). Such duplexes hypothetically can initiate interference similar to eukaryotic microRNAs, and also contribute to the exoRNA stability. Among exoRNAs, fragments of tRNAs, rRNAs, mRNAs, and sRNAs can be found, that is rather expected. Interestingly in (Alikina et al., 2018) a big fraction of exoRNAs was represented by the fragments derived from previously unknown antisense RNAs. Being in line with the cases described in (Dauros-Singorenko et al., 2018) this observation focuses attention on the functional role of such regulatory molecules.

Antisense RNAs (aRNAs) seemed to have the most predictable mechanism of action, forming complementary duplexes with the mRNAs of adjacent or overlapping genes (Werner, 2013). Their genomic arrangement indicates that they might be part of self-regulatory circuits that allow genes to regulate their own expression. For example, antisense and divergent transcripts can transmit regulatory signals to neighbouring promoters (Pelechano and Steinmetz, 2013).

Despite this seeming simplicity, till now the mechanisms of aRNAs action are not very well understood. A series of genome-wide studies led to a suggestion that in prokaryotic genomes antisense transcripts are just by-products of noise without any significant functions (Lloréns-Rico et al., 2016). The comparative genomic analysis demonstrated that in contrast to intragenic promoters aimed to initiate synthesis of shortened products in sense direction, antisense promoters were weakly conserved among closely related bacteria, and function as “promoters” only due to high A/T content of their upstream regions that allow the RNA polymerase binding (Shao et al., 2014; Singh et al., 2014). In line with this, the conclusion was made that the divergent transcription which is rather widespread not only in eukaryotic, but also in bacterial and archaeal genomes is a consequence of sequence symmetry (Warman et al., 2021). However, taking into account the observation about the presence of aRNA fragments in the pool of secreted RNAs, we assumed that at least some of such transcripts might be functional.

One of the major exoRNAs detected in Alikina et al., 2018 was mapped on the *uxuR* gene coding for a transcriptional regulator of hexuronate metabolism. UxuR belongs to the GntR family of regulators with the helix-turn-helix N-terminal DNA binding domain and the C-terminal ligand binding/dimerization domain (Suvorova et al., 2011). Its main regulon involves genes coding for all key enzymes (*uxuA, uxuB, uidA, uxaC, uxaA*), transporters (*uidB, uidC, exuT, gntP*), and regulators (*uidR, uxuR, exuR)* of hexuronate metabolism (Ritzenthaler and Mata-Gilsinger, 1982; Bates Utz et al., 2004; Suvorova et al., 2011; Tutukina et al., 2016). However, comparison of transcriptomes of the wild type K-12 MG1655 and its *uxuR* deletion derivative revealed many more possible targets that at least partially could be due to regulatory RNAs synthesized from within the *uxuR* gene.

The aim of this study was to compare the ability of candidate intragenic promoters for synthesis of antisense and shortened sense transcripts in *Escherichia coli in silico*, *in vitro* and *in vivo*, to study intragenic promoters with ability to initiate synthesis of regulatory RNAs within the *uxuR* gene, and to assess specificity of regulons belonging to UxuR protein and to the *uxuR*-derived short RNA-products.

## 2 Materials and methods

### 2.1 Strains and growth conditions

*Escherichia coli* K-12 MG1655 strain (GenBank accession number: U00096.3) was used as the model organism. For RNA extraction, cells were grown to OD_650_ of 0.3–0.4 (exponential phase) under constant shaking at 37°С in Minimal Salts (MS) media supplemented with 5% LB, 0.2% D-glucuronic acid or 0.2% D-glucose. The *uxuR* gene disruption was made as described in (Datsenko and Wanner, 2000) and then transferred to K-12 MG1655 strain with P1 transduction. To switch the UxuR synthesis off, the ribosome binding site and starting ATG codon of the *uxuR* gene were deleted using Gene Doctoring (Lee et al., 2009).

### 2.2 Promoter mapping

Promoter-like motifs within the *E. coli* K12 MG1655 genome were found by the pattern-recognition software PlatProm (http://mathcell.ru/model6.php?l=en; Shavkunov et al., 2009). The scoring system of this software is based on position-dependent weight matrices. Besides conservative base pairs recognised by the major sigma 70 subunit of RNA polymerase (RNAP), it takes into account several other features of the promoter DNA considering it as a common platform for interaction with both RNAP and transcription regulators. Sensitivity of the program is 85,5% at the cut off level ensuring prediction of transcription signals with *p*–value equal to 0.00004.

To calculate correlations between the PlatProm scores and efficiency of RNAP binding, we analysed two ChIP-chip datasets made with σ^70^- RNAP (Herring et al., 2005; Reppas et al., 2006). Maximal log_2_(Cy5/Cy3) registered at ± 250 bp from the most prominent transcription start site was taken. We analysed only those antisense (*n* = 663) and co-directed (*n* = 241) promoters that cannot initiate transcription of adjacent genes and do not overlap with any of the known or predicted gene promoters.

### 2.3 Testing promoter activity *in vitro*

Ability of RNAP to interact with the predicted intragenic promoters was estimated by electrophoretic mobility shift assays (EMSA). DNA fragments were PCR amplified with primers indicated in Supplementary Table S1. Amplicons were then loaded on a 5% polyacrylamide gel and purified as described in (Maniatis et al., 1982). RNAP-promoter complexes were formed at 37°C in a standard buffer, containing 50 mM Tris-HCl (pH 8.0), 0.1 mM EDTA, 0.1 mM DTT, 10 mM MgCl_2_, 50 mM NaCl, BSA (5 mg/mL), 1 pmol of PCR-generated DNA-fragments and 2–8 pmol of σ^70^-RNA polymerase (Sigma, United States). Interaction was allowed for 30 min, then 20 μg/mL heparin was added to prevent any non-specific binding. Samples were loaded on a prewarmed gel under very low voltage. Gels were then stained with ethidium bromide, visualised under the UV-light and photographed.

To test the ability of promoters within the *uxuR* gene to form open complexes, potassium permanganate footprinting was used (Zaychikov et al., 1997). 0.2 pmol of ^32^P-labelled PCR-generated DNA-fragments were incubated with 0.4–0.8 pmol of σ^70^-RNA polymerase (Sigma, United States) as described above. Transcript starts for the RNAs synthesised from within the *uxuR* gene were localized by single round transcription *in vitro* as previously described (Ozoline et al., 2001). To assess the direction of RNA synthesis, DNA templates of different lengths were used. The products of both reactions were separated in denaturing 6% polyacrylamide gel. Gels were calibrated by the products of Maxam-Gilbert G-specific sequencing and standard ^32^P-labelled markers (New England Biolabs, United States).

### 2.4 RNA extraction and sequencing

Total RNA from the exponentially growing cells (4 h of growth) was isolated using the RNAqueous RNA isolation kit (Ambion, Thermo Scientific, United States) according to the manufacturer’s protocol. Sequencing libraries were prepared using the NEBNext Ultra II Directional RNA library prep kit for Illumina (New England Biolabs, United States). Sequencing was performed as 50 nt SE on the Illumina HiSeq 4,000 machine.

Fraction of small exoRNAs was isolated as described in (Alikina et al., 2018). In brief, 5 mL of bacterial culture grown till OD_650_ = 0.4 was centrifuged at 4,500 rpm + 4°С. Supernatant was filtered through two 0.22 μm PES filters and aliquoted 500 μL/1.5 mL Eppendorf tube. Then, equal volume of the TriZol reagent (Thermo Scientific, United States) was added to each tube, and RNAs were extracted using the miRNeasy SerumPlasma kit (Qiagen, Germany).

The RNA quality was checked using 4% polyacrylamide gel with 8 M urea, and quantity measured on a Qubit fluorometer (Thermo Scientific, United States). Sequencing libraries were prepared using the NEBNext Multiplex Small RNA Library Prep Kit for Illumina (New England Biolabs, United States). Sequencing was performed as 150 + 150 PE on the Illumina NextSeq 500 machine. Sequencing was performed at the Skoltech Core Genomics Facility.

### 2.5 Transcriptomic analysis

The RNA sequencing data was processed using the following pipeline. Fastq files first underwent quality analysis using the FastQC tool (https://www.bioinformatics.babraham.ac.uk/projects/fastqc/) and then were trimmed using the Trim_Galore! tool. (https://bmcbioinformatics.biomedcentral.com/articles/10.1186/s12859-016-0956-2). The rRNA sequences were removed using SortMeRNA (https://academic.oup.com/bioinformatics/article/28/24/3211/246053). The reads were then aligned to the *E. coli* genome using STAR-aligner (https://pubmed.ncbi.nlm.nih.gov/23104886/). The aligned reads were translated into counts using the featureCounts tool (https://pubmed.ncbi.nlm.nih.gov/24227677/). The differential expression analysis was carried out with the NOISeq tool (https://pubmed.ncbi.nlm.nih.gov/26184878/). Raw reads are available in NCBI GenBank (SRA) under accession PRJNA882437.

### 2.6 Analysis of exoRNA sequencing data

The quality of the sequencing data of secreted small RNAs was validated *via* FastQC, after which the reads were trimmed with FlexBar (https://github.com/seqan/flexbar). Next, the reads were aligned with Bowtie two and sorted with SAMtools, after which the number of reads per gene was calculated using featureCounts, which is a part of the Subread package (http://subread.sourceforge.net/). The featureCounts output count data normalisation (Trimmed Mean of M-values) was performed *via* the edgeR package (https://bioconductor.org/packages/release/bioc/html/edgeR.html) and then used as the input data for constructing a linear regression model and scatter plots. The regression models and scatter plots were implemented *via* the R programming language, and the ggplot2 packages (https://cran.rproject.org/web/packages/ggplot2/index.html) and ggrepel (https://cran.rproject.org/web/packages/ggrepel/index.html). Raw reads are available in NCBI GenBank (SRA) under accession PRJNA883224.

### 2.7 qRT-PCR

Cultures were grown till mid-exponential phase (4 h of growth) in the same conditions as had been used for RNA-seq. To test the influence of small RNAs on the gene expression, 1 μmol of artificially synthesised RNAs, UxuT (5′-CAA​GGG​UAA​ACG​UUC​CUU​GCG​CUU​UCU​UAA​AUU​AAG​AAG​UCG​CAA​UGA​GUA​UUA​CUU​UGU​AAA​UUG​CAG​GGU​AUU​GUU​U-3′), *uxuR*-aRNA (5′-UUU​AUC​CAG​CGG​CCA​UGA​AUC-3′), and *uxuR*-exoRNA (5′-ACU​CUU​UGC​GAU​ACA​GGC​UGU​C-3′, Synthol, Russia) were added to 10 mL of cultures. Total RNA was isolated using the TriZol reagent (Thermo Scientific, United States) and treated with DNAse I (Promega, United States) according to the manufacturers’ protocols. Reverse transcription was made using respective gene specific primers and RevertAid MMul-V reverse transcriptase (Fermentas, Thermo Scientific, Lithuania). A DT-Lite thermocycler (DNA-Technology, Russia) and qPCRmix-HS SYBR (Evrogen, Russia) were used for quantitative PCR (qRT-PCR). Primers used for reverse transcription and amplification were as follows: flgK_RT 5′-TTA​CTC​ACC​AGC​GTT​TGC​AG-3’; flgK_PCR 5′-CTG​GTG​TGC​AGC​GTG​AGT​AT-3’; fliA_RT 5′-GCG​TTG​CGG​CCA​AGT​TCC​TG-3’; fliA_PCR 5′-CTA​TGC​TGG​ATG​AAC​TTC​GCA-3’. No PCR products were detected in negative controls in the absence of reverse transcriptase. Data obtained from at least three biological samples and analysed in three statistical replicates were calculated by the ΔC_t_ method. The error bars indicate the standard deviations of the respective mean values.

### 2.8 Modelling of RNA interactions

Targets for sRNA binding were found using IntaRNA (Mann et al., 2017). UxuT was modelled with RNA structure (Xu and Mathews, 2016).

## 3 Results

### 3.1 Distribution of potential intragenic promoters in the *Escherichia coli* genome and their ability to bind RNA polymerase

Besides expected promoters located upstream of coding sequences, PlatProm predicted a large number of unexpected promoters within coding sequences (Tutukina et al., 2007; Shavkunov et al., 2009). If active, they may drive synthesis of either shortened mRNAs or antisense RNA-products. However, some of them may interact with RNAP without initiating transcription or represent false positives. To evaluate the ability of predicted promoters to bind RNA polymerase, we used two datasets: ChIP-on-chip data reflecting genome-wide distribution of RNAP binding sites *in vivo* (Herring et al., 2005; Reppas et al., 2006) and *in vitro* results of EMSA performed here.

**FIGURE 1:**
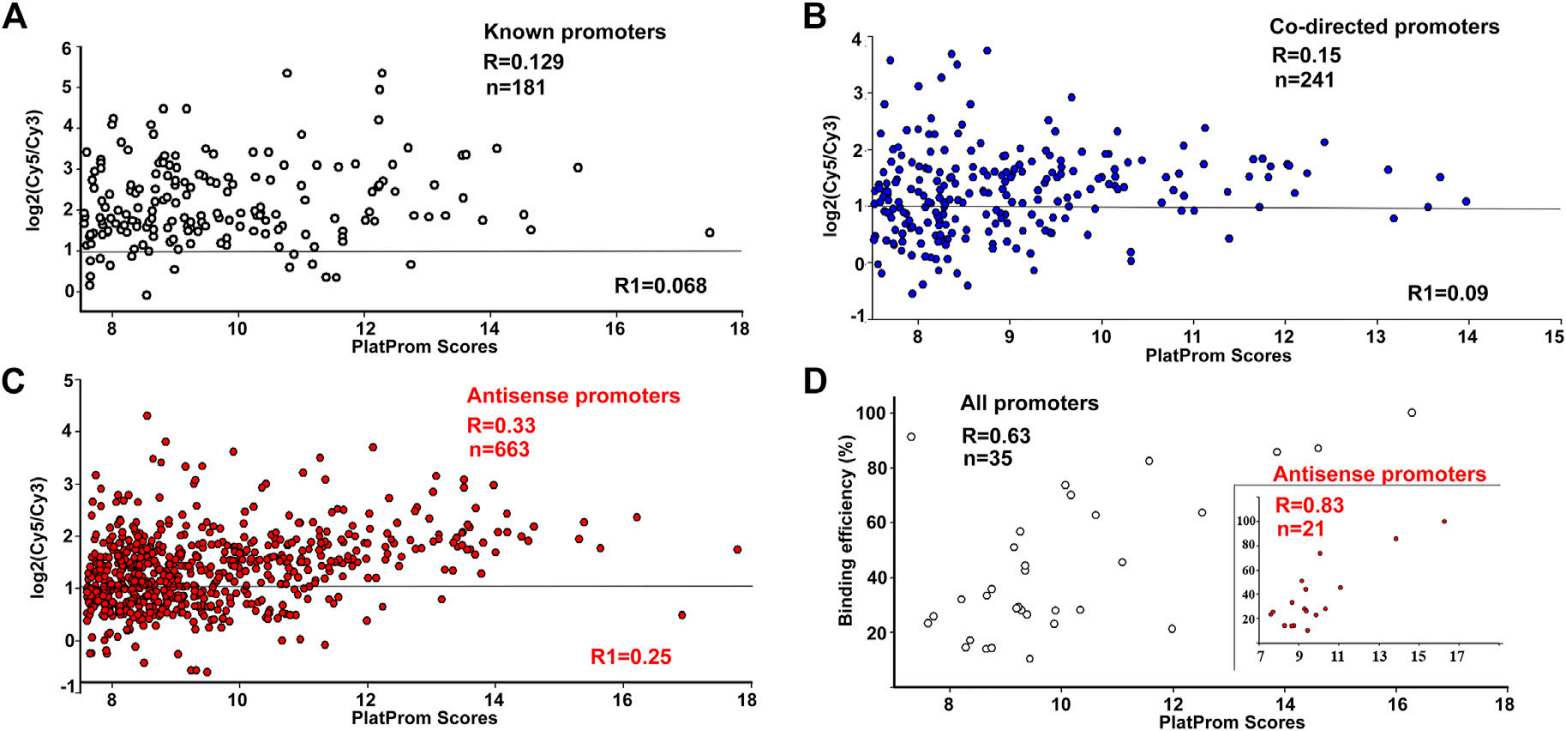
Correlation between promoter strength *in silico* (PlatProm score), and its ability to bind RNA polymerase *in vivo* **(A–C)** and *in vitro* **(D)**. ChIP-chip data for R are from (Herring et al., 2005), for R1, from (Reppas et al., 2006). Plots are shown only for the data from (Herring et al., 2005). Horizontal lines indicate the cut-off level for the ChIP-chip data.

According to ChIP-on-chip data, 92% of known, 77% of predicted upstream, and 75% of intragenic co-directed promoters were associated with the registered sites of RNA polymerase binding (cut off level was taken as log_2_(Cy5/Cy3) > 1, Figure 1), indicating a good correspondence between *in silico* and *in vivo* data. However, only 54% of putative antisense promoters were able to bind RNAP at the same cut off level (Figure 1C). This difference was not *a priori* expected and can indicate the orientation-dependent interference with elongating RNA polymerase in the living cells.

For promoter recognition, PlatProm uses known motifs of transcription factors and accounts for the presence of both direct and inverted repeats as potential targets for unknown regulators. Thus, the scores of promoters interacting with repressors are usually higher than their actual affinity to RNA polymerase, while RNAP-DNA complexes on weak promoters with low scores may be significantly stabilised by activators. As a result, no quantitative correlation with the PlatProm scores was observed for binding efficiency of “true” promoters (Figure 1A). For 214 co-directed promoters it was statistically significant only in the case of the first set of Chip-on-chip data (R = 0.15, *p* = 0.0198) (Figure 1B). However, both data sets show statistically significant correlations with *in silico* prediction for 663 antisense promoters (R = 0.33 and 0.25, *p* < 0.00001) (Figure 1C).

The enzyme binding capacity was then tested *in vitro* for 35 DNA fragments, containing intragenic promoters, of which 21 were antisense. Examples of all types of observed modes of interaction with RNAP are shown in Supplementary Figure S1; they included concentration-dependent (rrnB-P1, hns-P. uxuR co-directed and rcsA antisense) and concentration-independent (hns antisense) complexes, multiple (hns-P, uxuR antisense, rcsA antisense) and single (rrnB-P1, hns antisense) complexes. In all cases with multiple complexes, multiple promoters were predicted. The overall ability of antisense promoters to interact with polymerase appeared to be very similar to known and co-directed promoters. Only two intragenic promoters failed to interact with the enzyme of which one was antisense (predicted within the *lacZ* gene, Supplementary Figure S1). Thus, both types of unusual promoters can bind RNA polymerase. In contrast to ChIP-chip *in vivo,* in these experiments transcription complexes were formed in the absence of transcription factors or ligands which can affect RNA polymerase binding. As a result, statistically significant correlation with PlatProm scores was observed for all sets of promoters (Figure 1D). Surprisingly we observed that the correlation coefficient for the DNA fragments containing antisense promoters was higher than for the whole set of tested templates (R = 0.83, *p* < 0.00001, Figure 1D).

### 3.2 Short RNAs are synthesised in the 3′-terminal part of the *uxuR* gene

**FIGURE 2:**
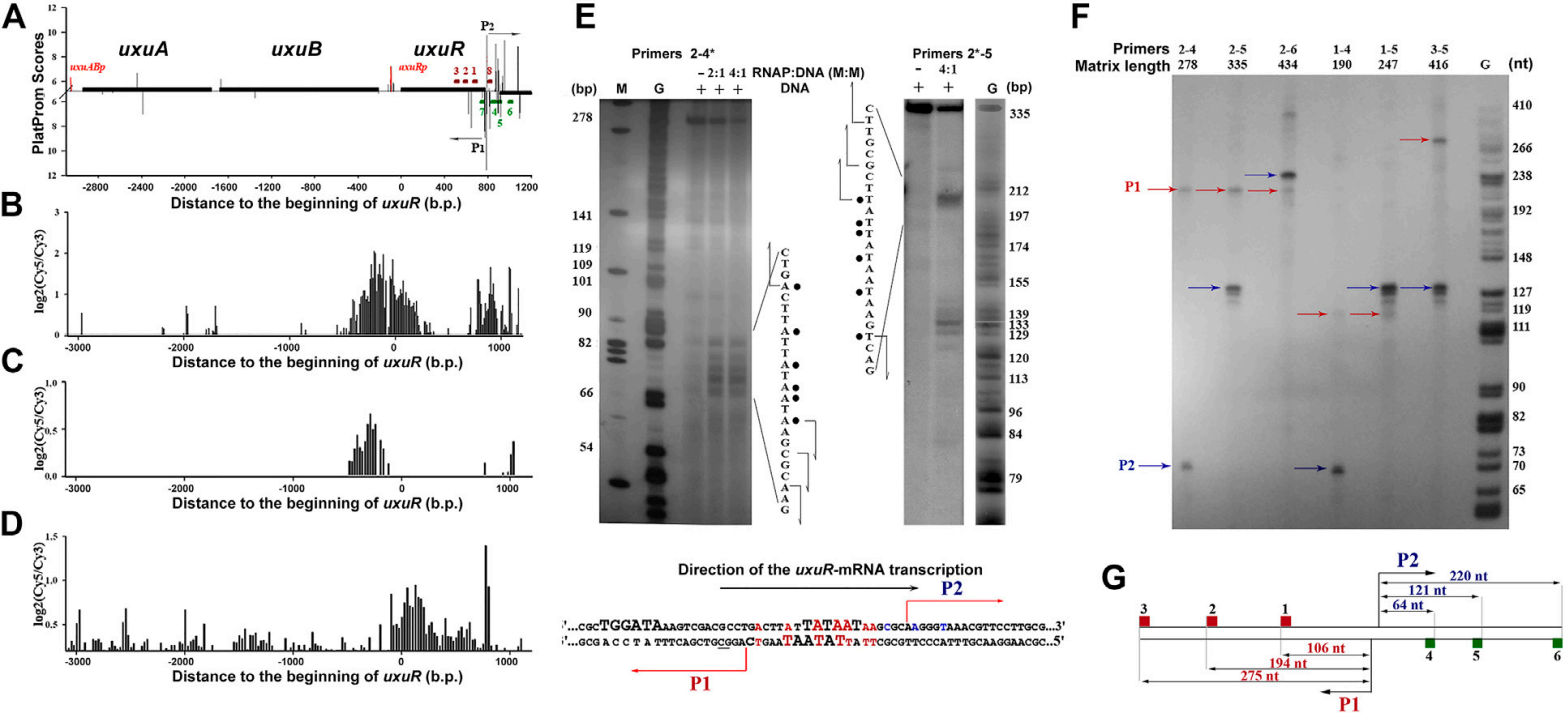
**(A)** Computational mapping reveals multiple promoters near the *uxuR* gene. Solid black lines above or below the *X*-axis show positioning and orientation of the genes. Bars represent promoters predicted on both strands. Oligonucleotides used in this work are shown by dark red and green rectangles (See Supplementary Table S2). **(B)** and **(C)** Distribution of the RNA polymerase binding sites in the *uxuR* region as estimated by the ChIP-chip technique (Herring et al., 2005; Reppas et al., 2006, respectively). **(D)** Expression efficiency data obtained in (Cold Spring Harbor Lab, 2023). **(E)** Permanganate footprinting of RNA polymerase complexes with the DNA fragment of the *uxuR* gene containing predicted promoters P1 and P2. Radioactive ^32^P-ATP was incorporated in the 5′-end of primer 4 (left gel) or primer 2 (right gel). Gels were calibrated with G-specific sequencing of the same fragments. The respective nucleotide sequences of each strand are shown. Modified thymines are marked with points. Predicted transcription start sites are indicated with arrows. Below is a schematic representation of P1 and P2 positioning in the *uxuR* sequence. −10 elements are set in bold capital letters. Positions modified by KMnO4 in a footprinting assay are shown in red. Positions from which transcription had started from P2 *in vitro* are shown in blue. **(F)** *In vitro* mapping of transcription starts for the small RNAs within the 3′-terminal part of the *uxuR* gene by single-round transcription assays. Primer pairs are indicated above the lanes. **(G)** Scheme showing the size of RNA products that should be detected from different templates if synthesis has started from P1 or P2.

One of the regions with high *in silico* scores for several predicted promoters (Figure 2A), which are tightly bound by RNA polymerase *in vitro* and *in vivo* and (Supplementary Figure S1, Figures 2B, C), and mediate the burst in the transcriptional output (Figure 2D) is located at the very end of the *uxuR* gene coding for a regulator of hexuronate metabolism (Figure 2A). The adjacent *yjiC* gene is transcribed in an opposite direction, thus the high density of promoter signals and their activity cannot be required for its expression.

*In vitro*, RNA polymerase formed at least two complexes with the fragment amplified with primers 2–5 (Supplementary Figure S1), and binding was detected in both ChIP-chip experiments (Figures 2B,C). The ability of the predicted promoters to form open complexes with RNA polymerase was confirmed by potassium permanganate footprinting (Figure 2E). According to positioning of modified thymines, these complexes can be assigned to divergent promoters P1 and P2 (Figures 2A, E).

To reveal direction of RNA synthesis in this region, a single-round assay with DNA templates amplified with different primer pairs was used (Figure 2F). Schematically, primers and resulting RNA products are shown in Figure 2G. If the 416 bp fragment amplified with pair three to five was used as a template, the products from at least four transcription start points, three of which located very closely to each other, were detected (Figure 2F). When the primer combination was changed to two to five, RNAs of about 120–130 nt were again detected, meaning that they were transcribed in the same direction as *uxuR.* Taking into account the 5%–7% difference in the mobility of RNA and DNA in the G-sequencing ladder, these RNAs exactly correspond to transcription initiation at P2, as confirmed by 5′-RACE (data not shown). Transcription from the antisense promoter was also detected giving a product of around 105–110 nt if the template was restricted by primer 1, around 195–200 nt for the templates with primer 2, and around 275–280 for the templates with primer 3. The respective product is initiated at the P1 promoter. Thus, both antisense P1 and co-directed P2 promoters are capable of productive transcription, with P2 being preferred by RNA polymerase in the studied conditions. Antisense promoters with lower scores located closer to primer 2 (Figure 2A) formed weaker open complexes with RNAP polymerase (Figure 2E, primers 2*-5) but were unable to initiate RNA synthesis.

### 3.3 Small RNAs synthesised from the 3′-terminal part of *uxuR* influence expression of genes related to the *Escherichia coli* motility

**FIGURE 3:**
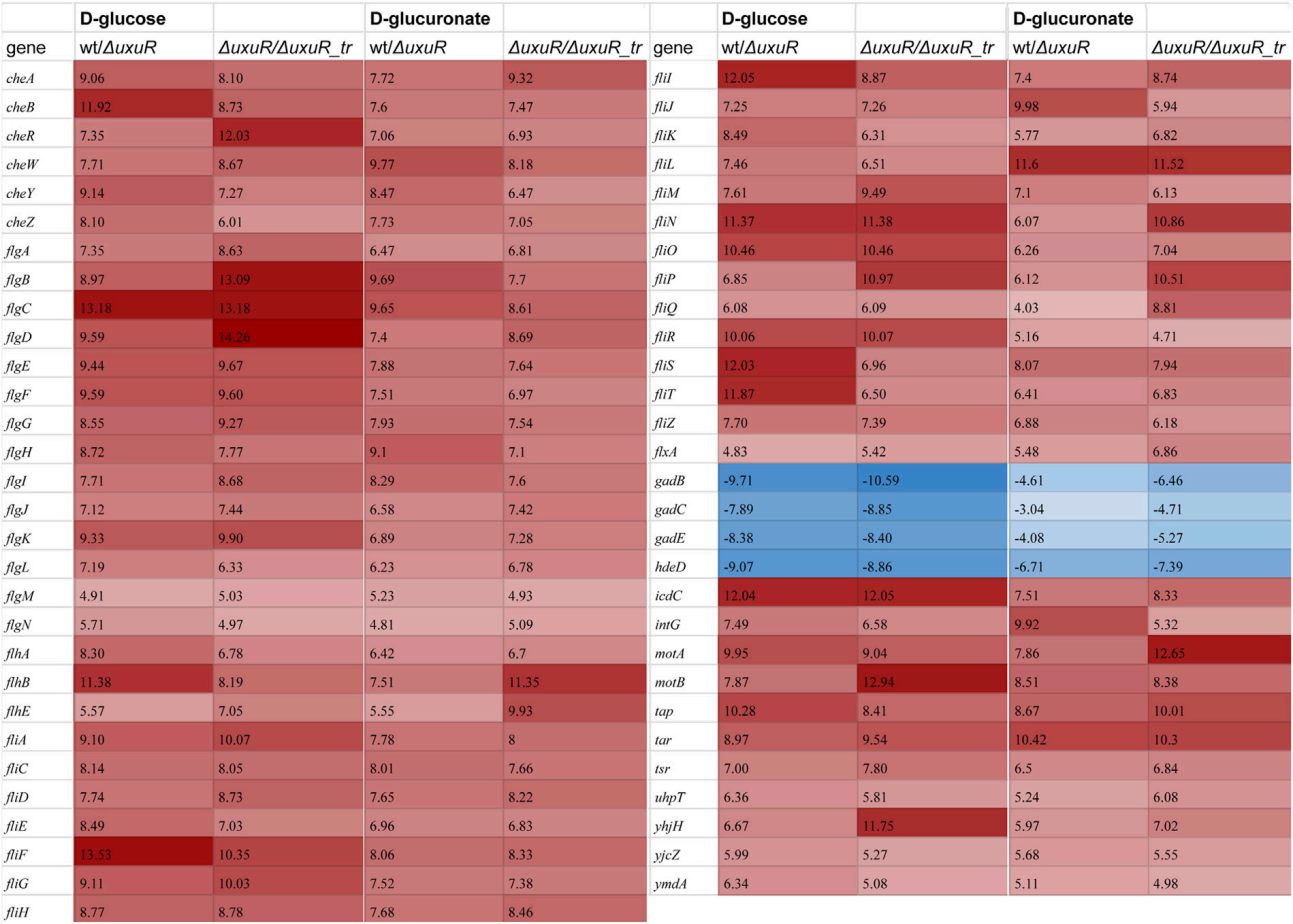
Heatmap demonstrating differential expression analysis for the wild type K-12 MG1655 (wt), its whole *uxuR* deletion derivative (*ΔuxuR),* and derivative with partial *uxuR* deletion only to switch protein synthesis off (*ΔuxuR_tr*). Values in the columns represent log_2_FC for the genes with significantly altered expression (*p*-value < 0.001). A full list of genes which were affected (*p*-value < 0.05) is shown in Supplementary Table S3. Red and blue colours indicate activation and repression, respectively. Growth conditions and compared strains are indicated in the column headers.

To check whether these RNAs play any role in the *E coli* cell, the transcriptomic analysis was performed for three strains: the wild type *E. coli* K-12 MG1655, the strain with deleted *uxuR* gene (*ΔuxuR* in Figure 3), and the strain where the fragment with the *uxuR*-mRNA translation start signals had been deleted (*ΔuxuR_*tr in Figure 3 *)*. In the latter case, the UxuR protein could not be produced, but all the possible promoters for small RNAs located closer to its end were still present. Keeping in mind that UxuR is a critical regulator of hexuronate metabolism, RNA sequencing was made for cultures growing on D-glucose or D-glucuronate, a key intermediate of the Ashwell pathway. On D-glucuronate, all genes coding for transporters, enzymes and regulators of hexuronate metabolism (*uxuAB, uxaCA, uxaB, exuT, gntP, uidABR, exuR*) were predictably activated, and the same was observed here and earlier on both carbon sources upon deletion of *uxuR* reflecting repressor function of its protein product (Tutukina et al., 2016).

More interestingly, for at least 59 genes expression critically changed upon deletion of whole *uxuR* and remained stable if small RNAs could be expressed from the end of the gene (wt/*ΔuxuR* and *ΔuxuR/ΔuxuR_*tr in Figure 3). This means that transcripts synthesised from within the *uxuR* gene (Figure 2) have a potential regulatory ability. When D-glucose had been used as a carbon source, the strongest changes (log_2_FC > 10) in the expression efficiency were detected for the genes of four operons linked with cell motility and chemotaxis, *flgBCDEFGHIJ*, *fliFGHIJK, fliDST and tar-tap-cheRBYZ*. They were all highly activated, suggesting that RNAs might act as their repressors, while genes responsive for cell resistance to extreme acid conditions were *vice versa* inhibited (*gadBS, gadE and hdeD*). Similar, albeit less pronounced changes were observed upon bacterial growth with D-glucuronate, indicating that the regulatory effects of the *uxuR* RNAs products could be not very much dependent on a carbon source.

**FIGURE 4:**
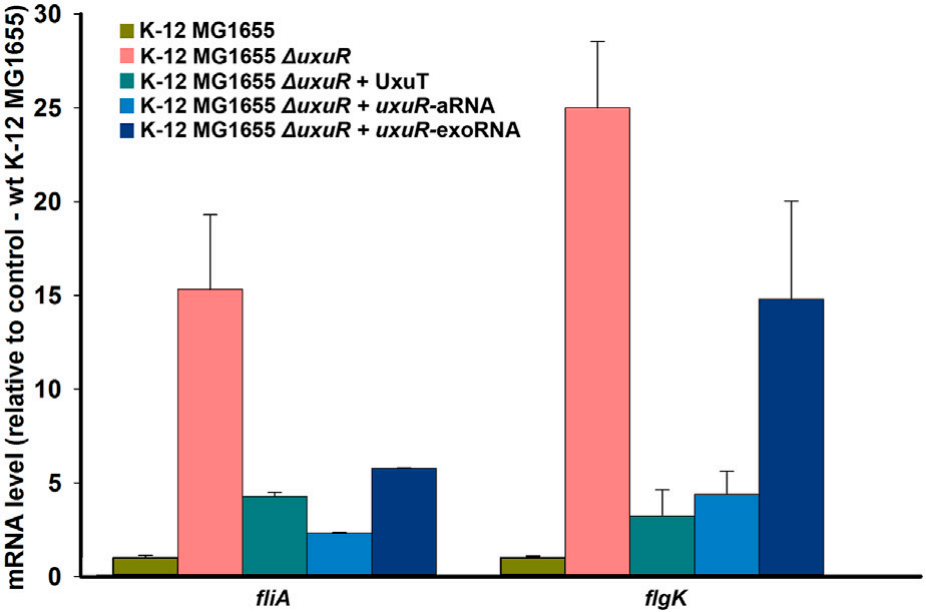
Expression dynamics of *fliA* and *flgK* upon deletion of *uxuR* and complementation with candidate regulatory RNAs. 1μmol of artificially synthesised RNAs were added to 10 mL of bacterial cultures. Strains are indicated above. Standard deviations were calculated based on three biological replicates, with four technical replicates in each.

To test whether the detected RNAs are indeed involved in the regulation of bacterial motility, their influence on the expression dynamics of the *fliA* gene coding for σ^28^ and thus controlling flagellar genes, and of *flgK* encoding structural flagellar hook-associated protein was checked. qRT-PCR was made with the RNAs isolated from the wild type K-12 MG1655, K-12 MG1655 *ΔuxuR* and K-12 MG1655 *ΔuxuR* with addition of one of three candidate RNAs: *uxuR*-aRNA synthesised from P1; co directed RNA UxuT (*uxuR* Terminator) synthesised from P2; and the most abundant secreted RNA (exoRNA) detected in (Alikina et al., 2018). RNA sequences are listed in Materials and Methods (2.7). Deletion of *uxuR* resulted in the activation of both *fliA* and *flgK* (pink bars in Figure 4). Addition of co-directed UxuT RNA (turquoise bars) and *uxuR*-aRNA (blue bars in Figure 4) significantly reduced this effect, suggesting that the *uxuR*-derived RNAs were indeed involved in regulation of bacterial motility. Addition of exoRNA also decreased the *fliA* activation compared to the *ΔuxuR* strain but had very little effect on *flgK*.

This exoRNA was one of the major RNAs detected in (Alikina et al., 2018). In general, in those experiments made on *E. coli* growing on minimal medium with D-glucose, huge amount of exoRNAs were mapped on the *uxuR* gene, including the fragment of *uxuR*-aRNA and its complementary region. Since UxuR controls hexuronate metabolism, which is connected to bacterial motility (Peekhaus and Conway, 1998), and D-galacturonic acid was later shown to regulate intestinal colonization by *E. coli* (Jimenez et al., 2020), it was reasonable to check what will happen to the profile and the amount of secreted RNAs upon change of the main carbon source to one of hexuronates.

### 3.4 Changes of the exoRNAs profile in *Escherichia coli* in response to growth with hexuronates

In (Alikina et al., 2018) it was shown that the number of secreted transcripts synthesised from the *uxuR* end significantly increased during co-cultivation with bacteria of the genus *Paenibacillus*. This indicated that the intragenic transcripts of *uxuR* could be specially produced for secretion, and under competition *E. coli* could export them as signalling molecules to adapt growth to a new environmental condition.

**FIGURE 5:**
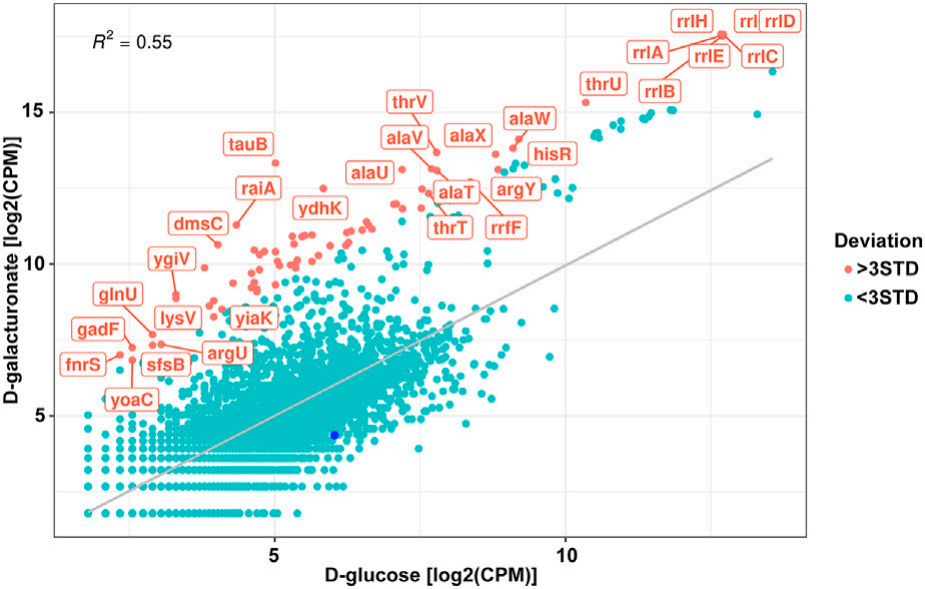
Correlation of the normalised read counts from the exoRNAs sequencing for the *Escherichia coli* K-12 MG1655 growing on D-glucose and D-galacturonate. Meanings deviated from the regression line for more than three STD are shown in red. Dark blue point represents log_2_FC for the reads mapped on *uxuR*.

A change of a carbon source significantly affected profiles of exoRNAs (Figure 5). Predictably, among genes to which altered numbers of exoRNAs were mapped were those coding for proteins involved in hexuronate metabolism, such as 2, 3-diketo-L-gulonate reductase YiaK, and transporters (*tauB*, *ydhK*). Among other affected genes were genes responsible for regulation of transcription (*sfsB* and *ygiV*) and translation (*raiA*), small RNAs FnrS and GadF, as well as rRNAs and tRNAs.

However, no significant increase or decrease in exoRNAs mapped to the terminal part of the *uxuR* gene was detected (dark blue point in Figure 5) indicating that they do not sense such a change in a carbon source. Since expression of *uxuR* itself depends on it (Ritzenthaler and Mata-Gilsinger, 1982; Tutukina et al., 2016) this means that studied transcripts can function independent of the protein in which gene they are encoded.

## 4 Discussion

Here, we have started with studying the features of intragenic promoters mapped by the promoter finder PlatProm in the *E. coli* K-12 MG1655 genome. PlatProms scores reflecting the presence of promoter-specific elements in the genomic loci were compared with the efficiency of RNA polymerase binding *in vivo* taken from ChIP-chip experiments and *in vitro* based on the percentage of the DNA bound in EMSA. Correlation of the PlatProm scores and the efficiency of RNAP binding *in vitro* for all tested promoter sets, known, co-directed and antisense, was rather high indicating that the algorithm predicts the strength of a binary complex formation to a certain extent. The highest correlation of 0.83 was detected for the set of DNA fragments containing potential antisense promoters, suggesting that the software can be specifically useful for their search.

Only 23 of 35 fragments tested *in vitro* were captured by RNA polymerase in ChIP-chip experiments *in vivo*, meaning that the ability of promoters to bind the enzyme may be to some extent unrealized in a bacterial cell. This happens mostly due to repressors occluding the RNAP binding to a promoter. Thus, the absence of correlation between the promoter scores *in silico* and their ability to bind RNA polymerase for both “true” promoters and intragenic co-directed ones was not unexpected. However, the correlation for the set of antisense promoters was statistically significant for both ChIP-chip experiments (Herring et al., 2005; Reppas et al., 2006; Figure 1B). This may be explained by the observation that antisense promoters usually lack additional promoter-specific elements but possess highly conserved hexanucleotides that mainly account for the RNA polymerase binding (Shao et al., 2014).

**FIGURE 6:**
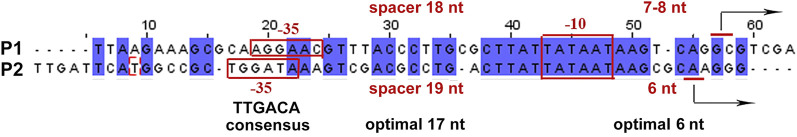
Alignment of sequences and comparison of regulatory elements in antisense (P1) and co-directed (P2) promoters located in the 3′-terminal part of *uxuR*.

Two divergent promoters, P1 and P2 (Figure 2), found at the very end of the *uxuR* gene represent an excellent example to study promoter interference due to the overlap of their −10 regions (Figure 2E; Figure 6). *In vitro* transcription assays revealed two products, with length corresponding to the assumed antisense (P1) and codirected (P2) promoters in the *uxuR* 3′-UTR (Figure 2F). They have almost similar PlatProm scores (9.35 and 9.86, respectively), but transcription from P2 is more efficient than could be expected given this difference. What could be the reasons for such a predominant choice of RNAP?

Both P1 and P2 have perfect −10 elements but due to the overlap RNA polymerase has to “decide”—which promoter is better. P2 has a more conservative −35 and an ideal spacer between −10 and +1, possessing an ideal dinucleotide CA at the transcription start point (Figure 6). This could be even more crucial *in vivo*; on the basis of several promoter studies done in our lab we can assume that the spacer length and dinucleotides in position + 1 are extremely significant for promoter work *in vivo* and even a 1 nt deviation may lead to a dramatic decrease of activity. According to classification of Akira Ishihama and others, these promoters are very likely constitutive (Shimada et al., 2014).

The absence of RNAs synthesised from within *uxuR* led to a dramatic increase in the expression of genes coding for flagellar assembly proteins (*fli*-and *flg-*operons), and those involved in chemotaxis (*motA, motB, fliM*) (Figure 3). The influence of the *uxuR* products on the expression of flagellar genes was further confirmed by qRT-PCR (Figure 4). Expression of *fliA* that encodes σ^28^ controlling all flagellar genes and *flgK* coding for a structural flagellar protein was 15 to 30-fold enhanced in the *uxuR* deletion mutant, and the effect was eliminated upon complementation with each of three sRNAs investigated here. The strongest suppression of both flagellar genes was observed upon addition of *uxuR*-aRNA and UxuT. When sequences of *uxuR*-aRNA and UxuT were aligned on the *E. coli* K-12 MG1655 genome to find suitable hybridization targets, *fliA* was one of them. To model possible interaction, IntaRNA was used (Mann et al., 2017). According to modelling results (Supplementry Figure S2), we may assume that *uxuR*-aRNA binds mRNA of *fliA* (at 2,001,070–2001819, lower strand) with energy of −12.33 kcal/mol, thus apparently affecting its stability. Also, this interaction might occur close to a putative transcription start at 2,001,544 (RegulonDB; marked in red in Supplementry Figure S2) and may influence transcription of *fliAZY*.

UxuT, in turn, may bind the promoter region strictly between the sites for FlhDC, an activator of *fliA*, and CsgD, serving as a repressor (Supplementry Figure S2). In contrast to *uxuR*-aRNA which has no secondary structure, UxuT has a stem-loop typical for sRNAs (Supplementry Figure S2). Due to the presence of non-canonical pairs in the stem-loop structure of its terminator, its hybridization energy is higher than that for the *fliA* target sequence (−6.7 kcal/mol *versus* −9.66 kcal/mol). As such, we can speculate that UxuT might interfere with FlhDC preventing this activator from binding and thus also inhibiting *fliA* expression. Further RIL-seq will be the best option to test if these interactions can indeed happen in the living cell.

The *uxuR* gene represents a nice example where three different types of transcripts with a regulatory potential were found: the 3′-UTR RNA UxuT transcribed from the promoter located in the rho-independent terminator of *uxuR*, aRNA transcribed divergently from the overlapping promoter, and exoRNA fragment produced from the antisense promoter located 148 bp upstream (Alikina et al., 2018). Our results suggest that it seems likely that all of them, to some extent, are involved in the regulation of bacterial motility. This is consistent with the recent paper about the crucial role of the UxuR structural homolog, ExuR, in colonization of the host organism and biofilm formation (Jimenez et al., 2020). Also, our observation about crucial difference in the expression of genes in K-12 MG1655 with deleted *uxuR* gene and switched off UxuR protein synthesis is in line with an earlier observation about a role of small RNAs in control of carbon metabolism and virulence in enteric bacteria (Papenfort and Vogel, 2014). On the other hand, it raises a question, whether all proteins whose functions have been revealed using expression analysis of a deletion mutant are indeed responsible for the detected changes, or there can be regulatory RNAs involved, that are encoded within the respective genes. In our case, due to the intragenic location of promoters for RNA synthesis it was not possible to delete or significantly weaken them without affecting the protein itself. Thus, the only option was to compare the whole-gene deletion mutant and the mutant where no protein is synthesised, but the gene is almost retained.

Interestingly, almost no genes coding for enzymes of hexuronate metabolism were affected by deletion of promoters for small RNAs, except *yiaK,* and almost no changes in their expression pattern were detected upon switching the carbon source (Figure 3). In line with this, no difference in the amount of the *uxuR*-derived exoRNAs was detected upon change from D-glucose to hexuronates (Figure 5). At the same time in (Alikina et al., 2018) it was clearly shown that the number of exoRNAs synthesised from the end of *uxuR* and their profile significantly changed in the presence of *Paenibacillus bisonicum* (PJFA00000000.1). These data suggest that small RNAs synthesised from within the *uxuR* gene, both intracellular and extracellular, might have specific functions unrelated to carbon metabolism. Many exoRNAs are synthesised from within genes coding for regulatory proteins that are not highly expressed themselves. We also noticed that they are often mapped to the genes overlapping with promoter regions of functional genes and small RNAs, suggesting some interplay with other regulatory elements of the bacterial genome.

In summary, although our findings are in line with a concept of widespread antisense transcription, they do not support the idea that bacterial aRNAs are simply the products of transcriptional noise. Both intracellular and secreted extracellular small RNAs may interfere with other regulatory events in bacterial cells, and their precise functions are yet to be understood.

## Data Availability

The datasets presented in this study can be found in online repositories. The names of the repository/repositories and accession number(s) can be found below: http://www.ncbi.nlm.nih.gov/bioproject/882437, PRJNA882437, http://www.ncbi.nlm.nih.gov/bioproject/883224, PRJNA883224.
