# Supplementary material for: Sense and antisense RNA products of the uxuR gene can affect motility and chemotaxis acting independent of the UxuR protein
Source: Front Mol Biosci. 2023 Feb 17;10:1121376. doi: 10.3389/fmolb.2023.1121376 (PMC10016265; doi:10.3389/fmolb.2023.1121376)
Supplement: Supplementary file 1 [file Table1.pdf]

Supplementary Table S3. List of genes demonstrating significant difference in expression levels for three strain types on two various carbon sources (glucuronate and glucose). WT represents wild type *E. coli* MG1655, DU has deleted *uxuR* gene, and in DUT only the UxuR protein expression is prevented, while all possible RNAs are transcribed from the respective locus. In the 2nd, 5th and 8th column the type of expression change is indicated (e.g. UP_wt_DOWN_du indicates that the respective gene expression is significantly higher in the wild type *E. coli* relative to DU type strain). LogFold columns shows the difference in expression levels, respective P-value is stated next to each. Cut-off P-value is 0.05.

Glucuronate									
**Gene**	**WT/DU**	**LogFold**	**P-value**	**WT/DUT**	**LogFold**	**P-value**	**DU/DUT**	**LogFold**	**P-value**
lysY	UP_wt_DOWN_du	-5,41	4,79E-03	-	-	-	UP_dut_DOWN_du	-4,11	3,77E-02
prpC	DOWN_wt_UP_du	3,85	1,86E-02	DOWN_wt_UP_dut	6,82	1,70E-04	UP_dut_DOWN_du	-2,96	4,38E-02
cspF	UP_wt_DOWN_du	-4,61	3,95E-03	-	-	-	UP_dut_DOWN_du	-3,27	3,07E-02
yjbI	-	-	-	-	-	-	UP_dut_DOWN_du	-6,89	2,54E-02
pagP	-	-	-	-	-	-	UP_dut_DOWN_du	-7,79	7,18E-03
gadE	UP_wt_DOWN_du	-4,08	1,86E-02	-	-	-	UP_dut_DOWN_du	-5,27	3,22E-03
metE	UP_wt_DOWN_du	-7,31	5,03E-05	UP_wt_DOWN_dut	-2,85	4,96E-02	UP_dut_DOWN_du	-4,45	5,70E-03
gadA	-	-	-	-	-	-	UP_dut_DOWN_du	-4,72	4,89E-03
gadB	UP_wt_DOWN_du	-4,61	8,30E-03	-	-	-	UP_dut_DOWN_du	-6,46	4,44E-04
gadC	UP_wt_DOWN_du	-3,04	4,24E-02	-	-	-	UP_dut_DOWN_du	-4,71	3,48E-03
gadX	UP_wt_DOWN_du	-4,44	5,86E-03	-	-	-	UP_dut_DOWN_du	-3,28	3,29E-02
proX	-	-	-	-	-	-	UP_dut_DOWN_du	-2,96	4,69E-02
proW	-	-	-	-	-	-	UP_dut_DOWN_du	-3,39	3,06E-02
proV	UP_wt_DOWN_du	-3,52	2,23E-02	-	-	-	UP_dut_DOWN_du	-4,33	6,34E-03
ymcE	UP_wt_DOWN_du	-5,92	6,94E-04	-	-	-	UP_dut_DOWN_du	-5,81	9,39E-04
dctR	-	-	-	-	-	-	UP_dut_DOWN_du	-3,57	4,48E-02
metY	UP_wt_DOWN_du	-3,50	1,98E-02	-	-	-	UP_dut_DOWN_du	-3,05	3,84E-02
hdeD	UP_wt_DOWN_du	-6,71	5,68E-04	-	-	-	UP_dut_DOWN_du	-7,39	1,99E-04
hdeB	UP_wt_DOWN_du	-3,05	4,09E-02	-	-	-	UP_dut_DOWN_du	-3,83	1,32E-02
hdeA	-	-	-	-	-	-	UP_dut_DOWN_du	-3,28	2,70E-02
pyrI	UP_wt_DOWN_du	-4,20	7,08E-03	-	-	-	UP_dut_DOWN_du	-3,16	3,40E-02
gltT	UP_wt_DOWN_du	-3,76	1,80E-02	-	-	-	UP_dut_DOWN_du	-3,49	2,98E-02
upp	UP_wt_DOWN_du	-4,31	6,36E-03	-	-	-	UP_dut_DOWN_du	-2,94	4,99E-02
cspH	UP_wt_DOWN_du	-7,61	1,25E-04	-	-	-	UP_dut_DOWN_du	-6,01	1,97E-03
cspI	UP_wt_DOWN_du	-3,59	1,77E-02	-	-	-	UP_dut_DOWN_du	-4,04	8,48E-03
carB	UP_wt_DOWN_du	-4,17	6,90E-03	-	-	-	UP_dut_DOWN_du	-3,99	9,05E-03
cspG	UP_wt_DOWN_du	-5,17	1,30E-03	-	-	-	UP_dut_DOWN_du	-5,31	1,02E-03
carA	UP_wt_DOWN_du	-4,69	3,24E-03	-	-	-	UP_dut_DOWN_du	-4,54	3,93E-03
cspB	UP_wt_DOWN_du	-3,49	1,90E-02	-	-	-	UP_dut_DOWN_du	-4,03	8,28E-03
bioF	UP_wt_DOWN_du	-5,99	1,86E-03	-	-	-	UP_dut_DOWN_du	-4,54	2,07E-02
gnsA	UP_wt_DOWN_du	-4,00	1,17E-02	-	-	-	UP_dut_DOWN_du	-3,33	3,59E-02
ymfI	UP_wt_DOWN_du	-6,42	4,72E-02	-	-	-	UP_dut_DOWN_du	-6,79	2,93E-02
cohE	UP_wt_DOWN_du	-8,84	1,28E-03	-	-	-	UP_dut_DOWN_du	-8,05	4,57E-03
yhiD	-	-	-	-	-	-	UP_dut_DOWN_du	-4,20	1,04E-02
rlmG	UP_wt_DOWN_du	-3,64	3,93E-02	-	-	-	UP_dut_DOWN_du	-3,35	4,39E-02
ydfK	UP_wt_DOWN_du	-5,39	5,00E-03	-	-	-	UP_dut_DOWN_du	-5,69	3,35E-03
uraA	UP_wt_DOWN_du	-4,85	3,22E-03	-	-	-	UP_dut_DOWN_du	-3,45	3,21E-02
arrS	UP_wt_DOWN_du	-3,98	4,16E-02	-	-	-	UP_dut_DOWN_du	-4,71	1,55E-02
fimA	UP_wt_DOWN_du	-2,92	4,49E-02	-	-	-	UP_dut_DOWN_du	-3,03	3,86E-02
pyrB	UP_wt_DOWN_du	-4,71	2,95E-03	-	-	-	UP_dut_DOWN_du	-3,64	1,59E-02
lit	UP_wt_DOWN_du	-7,91	5,67E-03	-	-	-	UP_dut_DOWN_du	-7,34	1,39E-02
ygiV	-	-	-	-	-	-	UP_dut_DOWN_du	-3,56	3,33E-02

paaZ	-	-	-	DOWN_wt_UP_dut	10,06	1,67E-04	UP_dut_DOWN_du	-4,67	4,95E-03
paaB	-	-	-	DOWN_wt_UP_dut	8,29	5,94E-05	UP_dut_DOWN_du	-5,44	1,52E-03
paaC	-	-	-	DOWN_wt_UP_dut	6,32	3,99E-04	UP_dut_DOWN_du	-5,91	7,92E-04
paaA	-	-	-	DOWN_wt_UP_dut	8,31	1,52E-05	UP_dut_DOWN_du	-5,35	1,05E-03
paaF	-	-	-	DOWN_wt_UP_dut	4,77	5,15E-03	UP_dut_DOWN_du	-4,10	1,31E-02
paaG	-	-	-	DOWN_wt_UP_dut	6,87	6,60E-04	UP_dut_DOWN_du	-3,44	2,83E-02
paaD	-	-	-	DOWN_wt_UP_dut	8,04	9,16E-05	UP_dut_DOWN_du	-5,18	2,30E-03
paaE	-	-	-	DOWN_wt_UP_dut	6,65	3,19E-04	UP_dut_DOWN_du	-4,69	4,06E-03
paaH	-	-	-	DOWN_wt_UP_dut	4,92	4,02E-03	UP_dut_DOWN_du	-4,25	1,03E-02
ymfD	UP_wt_DOWN_du	-8,57	2,02E-03	-	-	-	UP_dut_DOWN_du	-8,16	3,74E-03
ymfE	UP_wt_DOWN_du	-7,91	5,67E-03	-	-	-	UP_dut_DOWN_du	-7,97	5,26E-03
yhiM	-	-	-	-	-	-	UP_dut_DOWN_du	-3,49	3,70E-02
yedA	UP_wt_DOWN_du	-7,15	1,74E-02	-	-	-	UP_dut_DOWN_du	-6,97	2,22E-02
yhdV	DOWN_wt_UP_du	4,70	1,32E-02	-	-	-	DOWN_dut_UP_du	4,01	2,48E-02
yecT	-	-	-	-	-	-	DOWN_dut_UP_du	3,36	3,24E-02
yecR	DOWN_wt_UP_du	4,99	2,79E-03	-	-	-	DOWN_dut_UP_du	5,83	1,13E-03
ynjH	DOWN_wt_UP_du	3,71	2,30E-02	-	-	-	DOWN_dut_UP_du	4,85	9,34E-03
yhjH	DOWN_wt_UP_du	5,97	5,60E-04	-	-	-	DOWN_dut_UP_du	7,02	1,87E-04
yhjG	DOWN_wt_UP_du	3,92	1,16E-02	-	-	-	DOWN_dut_UP_du	3,78	1,42E-02
tsr	DOWN_wt_UP_du	6,50	1,77E-04	-	-	-	DOWN_dut_UP_du	6,84	1,08E-04
aer	DOWN_wt_UP_du	4,80	2,80E-03	-	-	-	DOWN_dut_UP_du	3,70	1,49E-02
ymdA	DOWN_wt_UP_du	5,11	2,38E-03	-	-	-	DOWN_dut_UP_du	4,98	2,68E-03
trg	DOWN_wt_UP_du	3,84	1,13E-02	-	-	-	DOWN_dut_UP_du	3,27	2,73E-02
uhpT	DOWN_wt_UP_du	5,24	1,84E-03	-	-	-	DOWN_dut_UP_du	6,08	7,48E-04
yjcZ	DOWN_wt_UP_du	5,68	9,04E-04	-	-	-	DOWN_dut_UP_du	5,55	1,01E-03
flhB	DOWN_wt_UP_du	7,51	1,28E-04	-	-	-	DOWN_dut_UP_du	11,35	1,86E-05
flhC	DOWN_wt_UP_du	4,82	2,98E-03	-	-	-	DOWN_dut_UP_du	3,98	1,04E-02
flhA	DOWN_wt_UP_du	6,42	2,54E-04	-	-	-	DOWN_dut_UP_du	6,70	1,50E-04
flhD	DOWN_wt_UP_du	4,55	4,44E-03	-	-	-	DOWN_dut_UP_du	3,36	2,69E-02
flhE	DOWN_wt_UP_du	5,55	2,48E-03	-	-	-	DOWN_dut_UP_du	9,93	2,06E-04
cheW	DOWN_wt_UP_du	9,77	5,60E-06	-	-	-	DOWN_dut_UP_du	8,18	2,58E-05
cheR	DOWN_wt_UP_du	7,06	1,95E-04	-	-	-	DOWN_dut_UP_du	6,93	2,19E-04
cheZ	DOWN_wt_UP_du	7,73	8,77E-05	-	-	-	DOWN_dut_UP_du	7,05	1,79E-04
cheY	DOWN_wt_UP_du	8,47	5,30E-05	-	-	-	DOWN_dut_UP_du	6,47	3,86E-04
cheB	DOWN_wt_UP_du	7,60	1,09E-04	-	-	-	DOWN_dut_UP_du	7,47	1,23E-04
cheA	DOWN_wt_UP_du	7,72	2,84E-05	-	-	-	DOWN_dut_UP_du	9,32	5,11E-06
yqjI	-	-	-	-	-	-	DOWN_dut_UP_du	3,14	4,64E-02
flxA	DOWN_wt_UP_du	5,48	1,31E-03	-	-	-	DOWN_dut_UP_du	6,86	3,43E-04
iraM	DOWN_wt_UP_du	5,02	1,55E-02	-	-	-	DOWN_dut_UP_du	7,95	5,26E-03
putP	DOWN_wt_UP_du	3,45	2,06E-02	-	-	-	DOWN_dut_UP_du	3,03	3,85E-02
putA	DOWN_wt_UP_du	4,59	4,33E-03	-	-	-	DOWN_dut_UP_du	4,30	6,17E-03
intG	DOWN_wt_UP_du	9,92	2,19E-04	-	-	-	DOWN_dut_UP_du	5,32	3,20E-03
icdC	DOWN_wt_UP_du	7,51	4,58E-05	-	-	-	DOWN_dut_UP_du	8,33	2,82E-05
fliZ	DOWN_wt_UP_du	6,88	1,35E-04	-	-	-	DOWN_dut_UP_du	6,18	3,63E-04
fliT	DOWN_wt_UP_du	6,41	5,87E-04	-	-	-	DOWN_dut_UP_du	6,83	3,66E-04
fliQ	DOWN_wt_UP_du	4,03	1,50E-02	-	-	-	DOWN_dut_UP_du	8,81	1,34E-03
fliP	DOWN_wt_UP_du	6,12	9,62E-04	-	-	-	DOWN_dut_UP_du	10,51	7,84E-05
fliS	DOWN_wt_UP_du	8,07	1,05E-04	-	-	-	DOWN_dut_UP_du	7,94	1,17E-04
fliR	DOWN_wt_UP_du	5,16	2,39E-03	-	-	-	DOWN_dut_UP_du	4,71	4,23E-03
fliM	DOWN_wt_UP_du	7,10	8,82E-05	-	-	-	DOWN_dut_UP_du	6,13	3,66E-04
fliL	DOWN_wt_UP_du	11,60	1,22E-05	-	-	-	DOWN_dut_UP_du	11,52	1,38E-05
fliO	DOWN_wt_UP_du	6,26	1,06E-03	-	-	-	DOWN_dut_UP_du	7,04	5,38E-04
fliN	DOWN_wt_UP_du	6,07	5,30E-04	-	-	-	DOWN_dut_UP_du	10,86	4,30E-05

.

fliI	DOWN_wt_UP_du	7,40	1,09E-04	-	-	-	DOWN_dut_UP_du	8,74	3,00E-05
fliH	DOWN_wt_UP_du	7,68	9,64E-05	-	-	-	DOWN_dut_UP_du	8,46	4,84E-05
fliK	DOWN_wt_UP_du	5,77	7,81E-04	-	-	-	DOWN_dut_UP_du	6,82	2,62E-04
fliJ	DOWN_wt_UP_du	9,98	1,94E-04	-	-	-	DOWN_dut_UP_du	5,94	1,62E-03
fliE	DOWN_wt_UP_du	6,96	3,25E-04	-	-	-	DOWN_dut_UP_du	6,83	3,66E-04
fliD	DOWN_wt_UP_du	7,65	3,44E-05	-	-	-	DOWN_dut_UP_du	8,22	2,39E-05
fliG	DOWN_wt_UP_du	7,52	4,29E-05	-	-	-	DOWN_dut_UP_du	7,38	7,21E-05
fliF	DOWN_wt_UP_du	8,06	1,78E-05	-	-	-	DOWN_dut_UP_du	8,33	2,00E-05
fliA	DOWN_wt_UP_du	7,78	2,45E-05	-	-	-	DOWN_dut_UP_du	8,00	1,55E-05
fliC	DOWN_wt_UP_du	8,01	1,04E-05	-	-	-	DOWN_dut_UP_du	7,66	1,91E-05
crfC	DOWN_wt_UP_du	4,47	5,26E-03	-	-	-	DOWN_dut_UP_du	4,33	6,75E-03
tap	DOWN_wt_UP_du	8,67	1,23E-05	-	-	-	DOWN_dut_UP_du	10,01	3,38E-06
tar	DOWN_wt_UP_du	10,42	1,82E-06	-	-	-	DOWN_dut_UP_du	10,30	2,04E-06
motA	DOWN_wt_UP_du	7,86	2,52E-05	-	-	-	DOWN_dut_UP_du	12,65	1,99E-06
motB	DOWN_wt_UP_du	8,51	2,31E-05	-	-	-	DOWN_dut_UP_du	8,38	2,60E-05
ycgR	DOWN_wt_UP_du	3,18	3,90E-02	-	-	-	DOWN_dut_UP_du	4,86	3,35E-03
yedN	DOWN_wt_UP_du	7,92	5,67E-03	-	-	-	DOWN_dut_UP_du	7,85	6,62E-03
flgC	DOWN_wt_UP_du	9,65	6,88E-06	-	-	-	DOWN_dut_UP_du	8,61	1,73E-05
flgB	DOWN_wt_UP_du	9,69	6,47E-06	-	-	-	DOWN_dut_UP_du	7,70	4,80E-05
flgA	DOWN_wt_UP_du	6,47	2,37E-04	-	-	-	DOWN_dut_UP_du	6,81	1,90E-04
flgG	DOWN_wt_UP_du	7,93	2,13E-05	-	-	-	DOWN_dut_UP_du	7,54	3,57E-05
flgF	DOWN_wt_UP_du	7,51	4,19E-05	-	-	-	DOWN_dut_UP_du	6,97	8,99E-05
flgE	DOWN_wt_UP_du	7,88	1,63E-05	-	-	-	DOWN_dut_UP_du	7,64	2,63E-05
flgD	DOWN_wt_UP_du	7,40	3,71E-05	-	-	-	DOWN_dut_UP_du	8,69	8,72E-06
flgK	DOWN_wt_UP_du	6,89	8,92E-05	-	-	-	DOWN_dut_UP_du	7,28	5,15E-05
flgJ	DOWN_wt_UP_du	6,58	2,04E-04	-	-	-	DOWN_dut_UP_du	7,42	9,49E-05
flgI	DOWN_wt_UP_du	8,29	3,39E-05	-	-	-	DOWN_dut_UP_du	7,60	6,91E-05
flgH	DOWN_wt_UP_du	9,10	1,80E-05	-	-	-	DOWN_dut_UP_du	7,10	1,33E-04
flgN	DOWN_wt_UP_du	4,81	2,68E-03	-	-	-	DOWN_dut_UP_du	5,09	1,98E-03
flgM	DOWN_wt_UP_du	5,23	1,89E-03	-	-	-	DOWN_dut_UP_du	4,93	2,73E-03
flgL	DOWN_wt_UP_du	6,23	2,52E-04	-	-	-	DOWN_dut_UP_du	6,78	1,16E-04
evgA	UP_wt_DOWN_du	-3,20	3,18E-02	-	-	-	-	-	-
prpE	DOWN_wt_UP_du	7,14	5,02E-04	DOWN_wt_UP_dut	8,20	6,86E-05	-	-	-
prpB	DOWN_wt_UP_du	7,06	5,79E-04	DOWN_wt_UP_dut	9,86	3,97E-06	-	-	-
thiC	UP_wt_DOWN_du	-3,19	3,15E-02	UP_wt_DOWN_dut	-3,33	2,55E-02	-	-	-
ypfM	DOWN_wt_UP_du	4,78	3,11E-03	DOWN_wt_UP_dut	2,98	4,88E-02	-	-	-
ugpB	DOWN_wt_UP_du	3,29	2,76E-02	-	-	-	-	-	-
rclR	DOWN_wt_UP_du	7,39	1,25E-02	-	-	-	-	-	-
fadB	DOWN_wt_UP_du	4,32	5,32E-03	DOWN_wt_UP_dut	4,34	5,15E-03	-	-	-
evgS	UP_wt_DOWN_du	-3,35	2,49E-02	-	-	-	-	-	-
prpR	DOWN_wt_UP_du	3,38	3,35E-02	-	-	-	-	-	-
yecJ	UP_wt_DOWN_du	-3,62	1,95E-02	-	-	-	-	-	-
yjiY	UP_wt_DOWN_du	-3,93	1,39E-02	UP_wt_DOWN_dut	-3,99	1,15E-02	-	-	-
fadD	DOWN_wt_UP_du	4,93	2,01E-03	DOWN_wt_UP_dut	3,99	9,12E-03	-	-	-
exbB	UP_wt_DOWN_du	-3,39	2,29E-02	-	-	-	-	-	-
fadI	DOWN_wt_UP_du	3,67	1,53E-02	DOWN_wt_UP_dut	4,30	5,84E-03	-	-	-
fadH	DOWN_wt_UP_du	4,90	2,59E-03	DOWN_wt_UP_dut	4,72	3,33E-03	-	-	-
yncE	UP_wt_DOWN_du	-4,15	7,00E-03	UP_wt_DOWN_dut	-3,09	3,50E-02	-	-	-
lhgO	DOWN_wt_UP_du	4,51	5,89E-03	-	-	-	-	-	-
codB	UP_wt_DOWN_du	-3,61	1,75E-02	-	-	-	-	-	-
fiu	UP_wt_DOWN_du	-7,20	5,74E-05	UP_wt_DOWN_dut	-5,82	4,79E-04	-	-	-
setB	UP_wt_DOWN_du	-3,62	3,61E-02	-	-	-	-	-	-
fadE	DOWN_wt_UP_du	3,39	2,32E-02	DOWN_wt_UP_dut	4,08	7,91E-03	-	-	-

.

ibsA	DOWN_wt_UP_du	3,32	3,12E-02	-	-	-	-	-	-	
thiE	UP_wt_DOWN_du	-3,56	2,12E-02	UP_wt_DOWN_dut	-2,93	4,86E-02	-	-	-	
metF	UP_wt_DOWN_du	-6,34	3,46E-04	UP_wt_DOWN_dut	-6,11	3,77E-04	-	-	-	
rrfE	UP_wt_DOWN_du	-6,64	3,99E-02	-	-	-	-	-	-	
lsrA	DOWN_wt_UP_du	5,87	6,47E-04	DOWN_wt_UP_dut	5,26	1,90E-03	-	-	-	
tnaA	DOWN_wt_UP_du	3,76	1,27E-02	DOWN_wt_UP_dut	3,19	3,04E-02	-	-	-	
lsrC	DOWN_wt_UP_du	5,74	9,27E-04	DOWN_wt_UP_dut	5,12	3,35E-03	-	-	-	
lsrB	DOWN_wt_UP_du	6,46	2,75E-04	DOWN_wt_UP_dut	5,15	3,18E-03	-	-	-	
aspU	UP_wt_DOWN_du	-4,02	2,07E-02	-	-	-	-	-	-	
lsrD	DOWN_wt_UP_du	3,92	1,49E-02	DOWN_wt_UP_dut	3,25	3,47E-02	-	-	-	
lsrG	DOWN_wt_UP_du	5,33	1,75E-03	DOWN_wt_UP_dut	3,90	1,99E-02	-	-	-	
lsrF	DOWN_wt_UP_du	5,09	1,84E-03	DOWN_wt_UP_dut	3,51	2,27E-02	-	-	-	
lsrK	DOWN_wt_UP_du	3,90	1,14E-02	DOWN_wt_UP_dut	3,73	1,58E-02	-	-	-	
leuV	UP_wt_DOWN_du	-5,58	1,73E-03	-	-	-	-	-	-	
leuW	UP_wt_DOWN_du	-3,29	2,80E-02	-	-	-	-	-	-	
leuT	UP_wt_DOWN_du	-4,89	2,99E-03	-	-	-	-	-	-	
lsrR	DOWN_wt_UP_du	4,62	4,07E-03	DOWN_wt_UP_dut	4,80	2,87E-03	-	-	-	
uidC	DOWN_wt_UP_du	7,20	5,27E-05	DOWN_wt_UP_dut	5,55	8,41E-04	-	-	-	
uidA	DOWN_wt_UP_du	6,30	1,93E-04	DOWN_wt_UP_dut	5,69	5,45E-04	-	-	-	
rrfC	UP_wt_DOWN_du	-3,09	4,34E-02	-	-	-	-	-	-	
puuA	-	-	-	DOWN_wt_UP_dut	2,99	4,19E-02	-	-	-	
malE	DOWN_wt_UP_du	3,28	2,64E-02	-	-	-	-	-	-	
leuZ	UP_wt_DOWN_du	-4,47	5,10E-03	-	-	-	-	-	-	
ydjO	UP_wt_DOWN_du	-3,85	2,61E-02	-	-	-	-	-	-	
asnA	UP_wt_DOWN_du	-3,11	3,51E-02	UP_wt_DOWN_dut	-3,17	3,17E-02	-	-	-	
ymdF	DOWN_wt_UP_du	4,69	4,73E-03	-	-	-	-	-	-	
tnaC	DOWN_wt_UP_du	4,24	7,48E-03	DOWN_wt_UP_dut	3,48	2,32E-02	-	-	-	
ymdB	DOWN_wt_UP_du	3,45	2,30E-02	-	-	-	-	-	-	
ryhB	UP_wt_DOWN_du	-3,07	3,96E-02	-	-	-	-	-	-	
yjcH	DOWN_wt_UP_du	3,76	1,39E-02	-	-	-	-	-	-	
metU	UP_wt_DOWN_du	-3,61	4,12E-02	-	-	-	-	-	-	
fhuE	UP_wt_DOWN_du	-4,07	9,53E-03	-	-	-	-	-	-	
metV	UP_wt_DOWN_du	-3,52	4,76E-02	-	-	-	-	-	-	
ytfJ	DOWN_wt_UP_du	3,35	2,57E-02	-	-	-	-	-	-	
araF	DOWN_wt_UP_du	5,06	2,46E-03	DOWN_wt_UP_dut	3,18	4,82E-02	-	-	-	
ybdL	UP_wt_DOWN_du	-4,17	8,89E-03	UP_wt_DOWN_dut	-4,61	4,52E-03	-	-	-	
mtfA	DOWN_wt_UP_du	2,92	4,54E-02	-	-	-	-	-	-	
yhhX	UP_wt_DOWN_du	-3,42	2,24E-02	UP_wt_DOWN_dut	-3,18	3,21E-02	-	-	-	
ygjR	DOWN_wt_UP_du	2,92	4,77E-02	-	-	-	-	-	-	
ychH	DOWN_wt_UP_du	2,88	4,80E-02	-	-	-	-	-	-	
tyrP	UP_wt_DOWN_du	-6,11	3,38E-04	UP_wt_DOWN_dut	-3,73	1,36E-02	-	-	-	
tyrV	UP_wt_DOWN_du	-5,18	6,91E-03	-	-	-	-	-	-	
malK	DOWN_wt_UP_du	3,02	4,22E-02	-	-	-	-	-	-	
ybiX	UP_wt_DOWN_du	-5,26	2,54E-03	UP_wt_DOWN_dut	-4,07	1,10E-02	-	-	-	
ynaE	UP_wt_DOWN_du	-4,08	1,39E-02	-	-	-	-	-	-	
aldB	DOWN_wt_UP_du	5,24	1,47E-03	DOWN_wt_UP_dut	4,02	1,00E-02	-	-	-	
metK	UP_wt_DOWN_du	-2,93	4,62E-02	-	-	-	-	-	-	
fepC	UP_wt_DOWN_du	-3,25	3,85E-02	-	-	-	-	-	-	
fepA	UP_wt_DOWN_du	-6,62	1,32E-04	UP_wt_DOWN_dut	-4,42	4,58E-03	-	-	-	
gspM	UP_wt_DOWN_du	-6,42	4,72E-02	-	-	-	-	-	-	
fepE	UP_wt_DOWN_du	-4,08	1,86E-02	-	-	-	-	-	-	
entA	UP_wt_DOWN_du	-4,05	9,68E-03	UP_wt_DOWN_dut	-3,52	2,01E-02	-	-	-	
entC	UP_wt_DOWN_du	-5,16	1,74E-03	UP_wt_DOWN_dut	-3,38	2,41E-02	-	-	-	

.

entB	UP_wt_DOWN_du	-6,07	6,50E-04	UP_wt_DOWN_dut	-4,10	8,61E-03	-	-	-
entD	UP_wt_DOWN_du	-6,64	2,96E-04	UP_wt_DOWN_dut	-4,94	2,49E-03	-	-	-
nrdF	UP_wt_DOWN_du	-3,17	3,59E-02	-	-	-	-	-	-
entF	UP_wt_DOWN_du	-5,66	6,52E-04	UP_wt_DOWN_dut	-4,39	5,05E-03	-	-	-
entH	UP_wt_DOWN_du	-4,57	4,41E-03	UP_wt_DOWN_dut	-3,61	1,82E-02	-	-	-
yeaH	DOWN_wt_UP_du	3,60	1,82E-02	-	-	-	-	-	-
sibA	DOWN_wt_UP_du	3,22	3,31E-02	-	-	-	-	-	-
metB	UP_wt_DOWN_du	-3,33	2,81E-02	UP_wt_DOWN_dut	-3,79	1,58E-02	-	-	-
kdgT	DOWN_wt_UP_du	3,94	1,03E-02	-	-	-	-	-	-
metA	UP_wt_DOWN_du	-3,61	2,00E-02	UP_wt_DOWN_dut	-3,86	1,66E-02	-	-	-
yccM	UP_wt_DOWN_du	-3,46	3,46E-02	-	-	-	-	-	-
ybdM	UP_wt_DOWN_du	-4,04	3,77E-02	-	-	-	-	-	-
fadL	DOWN_wt_UP_du	3,57	1,75E-02	DOWN_wt_UP_dut	3,17	3,17E-02	-	-	-
metN	UP_wt_DOWN_du	-3,25	3,69E-02	UP_wt_DOWN_dut	-3,25	3,53E-02	-	-	-
ytfK	DOWN_wt_UP_du	3,43	2,13E-02	-	-	-	-	-	-
fecI	UP_wt_DOWN_du	-4,82	2,87E-03	-	-	-	-	-	-
uidB	DOWN_wt_UP_du	6,16	2,67E-04	DOWN_wt_UP_dut	5,16	1,43E-03	-	-	-
bglJ	UP_wt_DOWN_du	-3,87	1,14E-02	UP_wt_DOWN_dut	-3,03	3,94E-02	-	-	-
yqeC	DOWN_wt_UP_du	3,36	3,76E-02	-	-	-	-	-	-
hokB	DOWN_wt_UP_du	3,08	4,37E-02	-	-	-	-	-	-
bglF	UP_wt_DOWN_du	-3,45	2,61E-02	-	-	-	-	-	-
sodB	DOWN_wt_UP_du	3,78	1,25E-02	DOWN_wt_UP_dut	3,13	3,41E-02	-	-	-
slp	UP_wt_DOWN_du	-3,32	4,39E-02	-	-	-	-	-	-
aspA	DOWN_wt_UP_du	3,29	2,57E-02	-	-	-	-	-	-
yddA	-	-	-	UP_wt_DOWN_dut	-3,56	2,65E-02	-	-	-
yjjZ	UP_wt_DOWN_du	-6,18	3,03E-04	UP_wt_DOWN_dut	-4,37	5,18E-03	-	-	-
rttR	UP_wt_DOWN_du	-7,35	1,39E-02	-	-	-	-	-	-
prpD	DOWN_wt_UP_du	4,64	5,69E-03	DOWN_wt_UP_dut	6,84	1,89E-04	-	-	-
yfhL	UP_wt_DOWN_du	-3,61	2,87E-02	-	-	-	-	-	-
ybdZ	UP_wt_DOWN_du	-4,37	2,42E-02	UP_wt_DOWN_dut	-3,68	4,16E-02	-	-	-
argQ	UP_wt_DOWN_du	-3,95	2,19E-02	-	-	-	-	-	-
ddpA	DOWN_wt_UP_du	3,52	2,87E-02	-	-	-	-	-	-
efeU	UP_wt_DOWN_du	-3,14	3,46E-02	-	-	-	-	-	-
argT	DOWN_wt_UP_du	3,41	2,19E-02	-	-	-	-	-	-
argV	UP_wt_DOWN_du	-3,73	2,74E-02	-	-	-	-	-	-
fimC	UP_wt_DOWN_du	-3,16	4,37E-02	-	-	-	-	-	-
acs	DOWN_wt_UP_du	4,17	6,60E-03	DOWN_wt_UP_dut	3,03	3,81E-02	-	-	-
efeO	UP_wt_DOWN_du	-3,40	2,40E-02	-	-	-	-	-	-
cnu	UP_wt_DOWN_du	-3,85	2,61E-02	-	-	-	-	-	-
ttcC	UP_wt_DOWN_du	-6,91	2,54E-02	-	-	-	-	-	-
cspD	DOWN_wt_UP_du	3,71	1,37E-02	-	-	-	-	-	-
hisR	UP_wt_DOWN_du	-4,74	3,20E-03	-	-	-	-	-	-
valT	UP_wt_DOWN_du	-3,55	1,97E-02	-	-	-	-	-	-
bioB	UP_wt_DOWN_du	-3,69	2,04E-02	-	-	-	-	-	-
bioA	UP_wt_DOWN_du	-4,77	4,65E-03	-	-	-	-	-	-
tyrA	UP_wt_DOWN_du	-3,42	2,26E-02	UP_wt_DOWN_dut	-3,59	1,74E-02	-	-	-
argZ	UP_wt_DOWN_du	-4,70	7,15E-03	-	-	-	-	-	-
astA	DOWN_wt_UP_du	5,85	5,17E-04	DOWN_wt_UP_dut	5,17	1,61E-03	-	-	-
astB	DOWN_wt_UP_du	5,16	1,66E-03	DOWN_wt_UP_dut	4,39	5,71E-03	-	-	-
astC	DOWN_wt_UP_du	6,40	2,12E-04	DOWN_wt_UP_dut	5,68	6,62E-04	-	-	-
astD	DOWN_wt_UP_du	6,06	4,59E-04	DOWN_wt_UP_dut	5,14	1,74E-03	-	-	-
astE	DOWN_wt_UP_du	5,86	6,91E-04	DOWN_wt_UP_dut	4,90	3,87E-03	-	-	-
yjbJ	DOWN_wt_UP_du	3,02	4,00E-02	-	-	-	-	-	-

.

uxuB	DOWN_wt_UP_du	2,86	4,84E-02	-	-	-	-	-	-
yghX	DOWN_wt_UP_du	3,87	1,33E-02	-	-	-	-	-	-
lysQ	UP_wt_DOWN_du	-6,42	4,72E-02	-	-	-	-	-	-
thrV	UP_wt_DOWN_du	-4,63	1,66E-02	-	-	-	-	-	-
gabD	DOWN_wt_UP_du	4,05	1,03E-02	-	-	-	-	-	-
valW	UP_wt_DOWN_du	-4,32	2,63E-02	-	-	-	-	-	-
intE	UP_wt_DOWN_du	-7,15	1,74E-02	-	-	-	-	-	-
tnaB	DOWN_wt_UP_du	3,89	1,12E-02	DOWN_wt_UP_dut	4,10	8,06E-03	-	-	-
yjfN	-	-	-	DOWN_wt_UP_dut	4,46	5,20E-03	-	-	-
lysT	UP_wt_DOWN_du	-3,75	1,41E-02	-	-	-	-	-	-
ymfJ	UP_wt_DOWN_du	-6,42	4,72E-02	-	-	-	-	-	-
yjfZ	UP_wt_DOWN_du	-8,03	4,90E-03	-	-	-	-	-	-
ynfM	UP_wt_DOWN_du	-3,91	1,11E-02	UP_wt_DOWN_dut	-4,03	9,21E-03	-	-	-
yegP	DOWN_wt_UP_du	3,67	1,83E-02	-	-	-	-	-	-
ykfB	DOWN_wt_UP_du	3,49	2,39E-02	-	-	-	-	-	-
fes	UP_wt_DOWN_du	-3,50	2,38E-02	UP_wt_DOWN_dut	-3,22	3,71E-02	-	-	-
ytfQ	DOWN_wt_UP_du	4,60	3,79E-03	-	-	-	-	-	-
lysW	UP_wt_DOWN_du	-3,87	1,43E-02	-	-	-	-	-	-
cbrC	UP_wt_DOWN_du	-3,70	3,37E-02	-	-	-	-	-	-
exbD	UP_wt_DOWN_du	-3,11	3,56E-02	-	-	-	-	-	-
suhB	UP_wt_DOWN_du	-3,96	1,07E-02	-	-	-	-	-	-
puuD	-	-	-	DOWN_wt_UP_dut	2,89	4,90E-02	-	-	-
yjhC	DOWN_wt_UP_du	2,97	4,59E-02	-	-	-	-	-	-
treA	DOWN_wt_UP_du	3,20	3,69E-02	-	-	-	-	-	-
fadJ	DOWN_wt_UP_du	2,99	4,09E-02	-	-	-	-	-	-
xylF	DOWN_wt_UP_du	4,41	7,24E-03	-	-	-	-	-	-
yrbN	UP_wt_DOWN_du	-3,23	3,59E-02	-	-	-	-	-	-
yegX	UP_wt_DOWN_du	-3,83	2,40E-02	-	-	-	-	-	-
ycdT	UP_wt_DOWN_du	-3,34	4,23E-02	-	-	-	-	-	-
yahO	DOWN_wt_UP_du	4,16	7,35E-03	-	-	-	-	-	-
araA	DOWN_wt_UP_du	3,80	3,70E-02	-	-	-	-	-	-
proM	UP_wt_DOWN_du	-8,07	4,57E-03	-	-	-	-	-	-
yhdW	DOWN_wt_UP_du	5,14	4,77E-03	DOWN_wt_UP_dut	3,51	4,65E-02	-	-	-
bioD	UP_wt_DOWN_du	-3,74	3,16E-02	-	-	-	-	-	-
cirA	UP_wt_DOWN_du	-7,97	1,28E-05	UP_wt_DOWN_dut	-5,44	8,30E-04	-	-	-
mmuP	UP_wt_DOWN_du	-4,37	1,35E-02	UP_wt_DOWN_dut	-4,62	7,06E-03	-	-	-
ydcS	DOWN_wt_UP_du	5,62	9,53E-04	DOWN_wt_UP_dut	4,17	8,32E-03	-	-	-
fecA	UP_wt_DOWN_du	-4,05	1,15E-02	UP_wt_DOWN_dut	-3,31	2,89E-02	-	-	-
paaI	-	-	-	DOWN_wt_UP_dut	4,98	1,36E-02	-	-	-
opgE	UP_wt_DOWN_du	-3,54	1,94E-02	-	-	-	-	-	-
lamB	DOWN_wt_UP_du	3,29	2,63E-02	-	-	-	-	-	-
ryfD	DOWN_wt_UP_du	3,23	2,83E-02	-	-	-	-	-	-
aroF	UP_wt_DOWN_du	-4,65	3,26E-03	UP_wt_DOWN_dut	-3,79	1,22E-02	-	-	-
actP	DOWN_wt_UP_du	4,21	6,37E-03	DOWN_wt_UP_dut	3,19	3,09E-02	-	-	-
ryjA	DOWN_wt_UP_du	3,26	3,27E-02	-	-	-	-	-	-
mqo	UP_wt_DOWN_du	-3,56	1,82E-02	UP_wt_DOWN_dut	-3,87	1,09E-02	-	-	-
fecR	UP_wt_DOWN_du	-4,64	4,22E-03	UP_wt_DOWN_dut	-3,09	3,96E-02	-	-	-
rhlE	UP_wt_DOWN_du	-3,69	2,04E-02	-	-	-	-	-	-
paaK	-	-	-	DOWN_wt_UP_dut	3,08	3,82E-02	-	-	-
thiG	-	-	-	UP_wt_DOWN_dut	-2,99	5,00E-02	-	-	-
pinR	UP_wt_DOWN_du	-3,81	2,78E-02	-	-	-	-	-	-
agp	DOWN_wt_UP_du	3,17	3,15E-02	-	-	-	-	-	-
trpE	-	-	-	UP_wt_DOWN_dut	-3,35	3,37E-02	-	-	-

ybeL	DOWN_wt_UP_du	3,55	1,82E-02	-	-	-	-	-	-
bsmA	DOWN_wt_UP_du	3,13	4,14E-02	DOWN_wt_UP_dut	4,99	2,09E-03	-	-	-
ves	DOWN_wt_UP_du	4,52	1,77E-02	-	-	-	-	-	-
yhhQ	UP_wt_DOWN_du	-3,78	2,96E-02	-	-	-	-	-	-
pgaA	UP_wt_DOWN_du	-4,23	1,68E-02	-	-	-	-	-	-
ydcV	DOWN_wt_UP_du	4,31	8,68E-03	DOWN_wt_UP_dut	3,35	4,23E-02	-	-	-
glpA	DOWN_wt_UP_du	2,90	4,60E-02	-	-	-	-	-	-
ydcT	DOWN_wt_UP_du	5,31	3,63E-03	DOWN_wt_UP_dut	4,24	1,62E-02	-	-	-
argY	UP_wt_DOWN_du	-4,73	5,95E-03	-	-	-	-	-	-
azuC	UP_wt_DOWN_du	-9,44	4,80E-04	UP_wt_DOWN_dut	-3,29	3,87E-02	-	-	-
csiD	DOWN_wt_UP_du	5,58	1,06E-03	DOWN_wt_UP_dut	3,28	4,18E-02	-	-	-
metR	UP_wt_DOWN_du	-4,31	1,48E-02	UP_wt_DOWN_dut	-4,55	7,92E-03	-	-	-
patD	DOWN_wt_UP_du	3,77	1,37E-02	-	-	-	-	-	-
glpK	DOWN_wt_UP_du	2,85	4,89E-02	-	-	-	-	-	-
tynA	DOWN_wt_UP_du	3,64	2,36E-02	-	-	-	-	-	-
ytfT	DOWN_wt_UP_du	3,18	4,84E-02	-	-	-	-	-	-
gabT	DOWN_wt_UP_du	3,60	1,77E-02	-	-	-	-	-	-
mglB	DOWN_wt_UP_du	3,74	1,31E-02	DOWN_wt_UP_dut	2,89	4,76E-02	-	-	-
gabP	DOWN_wt_UP_du	4,05	1,32E-02	-	-	-	-	-	-
lysZ	UP_wt_DOWN_du	-4,34	9,35E-03	-	-	-	-	-	-
entE	UP_wt_DOWN_du	-5,21	1,65E-03	UP_wt_DOWN_dut	-4,12	8,22E-03	-	-	-
pyrD	UP_wt_DOWN_du	-3,96	1,08E-02	-	-	-	-	-	-
metW	UP_wt_DOWN_du	-4,05	8,88E-03	-	-	-	-	-	-
raiA	DOWN_wt_UP_du	3,43	2,09E-02	-	-	-	-	-	-
yjjQ	UP_wt_DOWN_du	-4,70	1,45E-02	UP_wt_DOWN_dut	-4,02	2,46E-02	-	-	-
yghD	UP_wt_DOWN_du	-4,63	1,66E-02	-	-	-	-	-	-
mhpR	DOWN_wt_UP_du	3,29	2,76E-02	-	-	-	-	-	-
nrdE	-	-	-	UP_wt_DOWN_dut	-3,09	3,71E-02	-	-	-
ansP	UP_wt_DOWN_du	-3,45	4,27E-02	-	-	-	-	-	-
ytfR	DOWN_wt_UP_du	3,33	3,01E-02	-	-	-	-	-	-
glpF	DOWN_wt_UP_du	3,12	3,31E-02	-	-	-	-	-	-

..

Glucose									
Gene	WT/DU	LogFold	P-value	WT/DUT	LogFold	P-value	DU/DUT	LogFold	P-value
ycgR	UP_du_DOWN_wt	-4,83	3,39E-03	-	-	-	UP_du_DOWN_dut	-4,84	3,35E-03
yncG	DOWN_du_UP_wt	4,97	1,27E-02	-	-	-	DOWN_du_UP_dut	4,63	2,23E-02
poxB	DOWN_du_UP_wt	3,37	2,46E-02	-	-	-	DOWN_du_UP_dut	3,83	1,20E-02
yecT	UP_du_DOWN_wt	-4,46	8,26E-03	-	-	-	UP_du_DOWN_dut	-4,47	8,05E-03
yncL	DOWN_du_UP_wt	3,29	4,64E-02	-	-	-	-	-	-
yecR	UP_du_DOWN_wt	-6,64	2,75E-04	-	-	-	UP_du_DOWN_dut	-5,93	6,50E-04
yciG	DOWN_du_UP_wt	9,14	7,86E-04	-	-	-	DOWN_du_UP_dut	8,91	1,14E-03
yciF	-	-	-	-	-	-	DOWN_du_UP_dut	4,40	3,13E-02
ydeI	DOWN_du_UP_wt	3,59	3,91E-02	-	-	-	DOWN_du_UP_dut	3,73	3,16E-02
sra	-	-	-	-	-	-	DOWN_du_UP_dut	2,92	4,50E-02
ynjH	UP_du_DOWN_wt	-3,40	3,65E-02	-	-	-	-	-	-
codB	-	-	-	-	-	-	UP_du_DOWN_dut	-4,01	1,36E-02
yhjH	UP_du_DOWN_wt	-6,67	2,11E-04	-	-	-	UP_du_DOWN_dut	-11,75	9,24E-06
codA	-	-	-	-	-	-	UP_du_DOWN_dut	-3,12	3,53E-02
mlrA	DOWN_du_UP_wt	4,11	1,82E-02	-	-	-	DOWN_du_UP_dut	3,73	3,16E-02
ycaC	DOWN_du_UP_wt	3,65	2,74E-02	-	-	-	DOWN_du_UP_dut	4,24	1,11E-02
yjbJ	-	-	-	-	-	-	DOWN_du_UP_dut	2,95	4,50E-02
hchA	DOWN_du_UP_wt	3,21	3,15E-02	-	-	-	DOWN_du_UP_dut	3,27	3,10E-02
cysP	-	-	-	UP_wt_DOWN_dut	-5,24	1,84E-03	-	-	-
tktB	DOWN_du_UP_wt	3,89	1,10E-02	-	-	-	DOWN_du_UP_dut	4,67	3,12E-03
yeaQ	DOWN_du_UP_wt	3,53	1,84E-02	-	-	-	-	-	-
tsr	UP_du_DOWN_wt	-7,00	8,99E-05	-	-	-	UP_du_DOWN_dut	-7,80	3,13E-05
katE	DOWN_du_UP_wt	4,20	8,32E-03	-	-	-	DOWN_du_UP_dut	4,61	4,24E-03
elaB	DOWN_du_UP_wt	3,46	2,09E-02	-	-	-	DOWN_du_UP_dut	3,27	2,80E-02
ygiV	-	-	-	-	-	-	DOWN_du_UP_dut	3,39	3,69E-02
osmC	-	-	-	-	-	-	DOWN_du_UP_dut	3,05	3,86E-02
osmE	-	-	-	-	-	-	DOWN_du_UP_dut	3,11	3,44E-02
pagP	DOWN_du_UP_wt	4,01	1,79E-02	-	-	-	DOWN_du_UP_dut	3,36	4,53E-02
gadE	DOWN_du_UP_wt	8,38	4,71E-05	-	-	-	DOWN_du_UP_dut	8,40	4,50E-05
yebV	DOWN_du_UP_wt	5,06	1,89E-03	-	-	-	DOWN_du_UP_dut	4,70	3,37E-03
metE	DOWN_du_UP_wt	4,69	2,85E-03	-	-	-	DOWN_du_UP_dut	4,62	3,14E-03
gadA	DOWN_du_UP_wt	6,94	1,29E-04	-	-	-	DOWN_du_UP_dut	7,43	5,53E-05
gadB	DOWN_du_UP_wt	9,71	4,82E-06	-	-	-	DOWN_du_UP_dut	10,59	1,04E-06
gadC	DOWN_du_UP_wt	7,89	1,93E-05	-	-	-	DOWN_du_UP_dut	8,85	3,69E-06
gcvH	-	-	-	-	-	-	UP_du_DOWN_dut	-3,87	2,48E-02
modA	UP_du_DOWN_wt	-3,12	3,68E-02	-	-	-	UP_du_DOWN_dut	-3,86	1,35E-02
modB	-	-	-	-	-	-	UP_du_DOWN_dut	-3,11	4,34E-02
aer	-	-	-	-	-	-	UP_du_DOWN_dut	-3,26	3,75E-02
gadX	DOWN_du_UP_wt	6,46	2,08E-04	-	-	-	DOWN_du_UP_dut	6,34	2,55E-04
gadY	-	-	-	-	-	-	DOWN_du_UP_dut	3,11	4,86E-02
osmY	DOWN_du_UP_wt	3,68	1,51E-02	-	-	-	DOWN_du_UP_dut	3,61	1,67E-02
ymdB	UP_du_DOWN_wt	-3,29	2,79E-02	-	-	-	-	-	-
gcvB	-	-	-	-	-	-	UP_du_DOWN_dut	-4,37	5,08E-03
gadW	DOWN_du_UP_wt	4,19	7,86E-03	-	-	-	DOWN_du_UP_dut	4,92	2,38E-03
pfkB	-	-	-	-	-	-	DOWN_du_UP_dut	3,41	2,83E-02
ydjO	-	-	-	UP_wt_DOWN_dut	-3,93	3,16E-02	-	-	-
ydjN	-	-	-	UP_wt_DOWN_dut	-3,28	2,85E-02	-	-	-
yjdJ	-	-	-	-	-	-	DOWN_du_UP_dut	3,55	3,15E-02
dps	DOWN_du_UP_wt	4,67	2,99E-03	-	-	-	DOWN_du_UP_dut	5,20	1,23E-03
ymdA	UP_du_DOWN_wt	-6,34	6,35E-04	-	-	-	UP_du_DOWN_dut	-5,08	2,68E-03
trg	-	-	-	-	-	-	UP_du_DOWN_dut	-3,39	2,65E-02

uhpT	UP_du_DOWN_wt	-6,36	6,18E-04	-	-	-	UP_du_DOWN_dut	-5,81	1,11E-03
wrbA	DOWN_du_UP_wt	3,70	1,49E-02	-	-	-	DOWN_du_UP_dut	3,78	1,40E-02
paoA	-	-	-	-	-	-	DOWN_du_UP_dut	6,50	4,72E-02
ybdK	DOWN_du_UP_wt	3,48	2,91E-02	-	-	-	DOWN_du_UP_dut	4,39	7,10E-03
yhcO	-	-	-	-	-	-	DOWN_du_UP_dut	4,03	1,74E-02
yodC	-	-	-	-	-	-	DOWN_du_UP_dut	3,48	3,24E-02
yjcZ	UP_du_DOWN_wt	-5,99	4,53E-04	-	-	-	UP_du_DOWN_dut	-5,27	1,32E-03
ynaE	DOWN_du_UP_wt	4,14	2,32E-02	-	-	-	-	-	-
flhB	UP_du_DOWN_wt	-11,38	1,74E-05	-	-	-	UP_du_DOWN_dut	-8,19	5,99E-05
flhC	UP_du_DOWN_wt	-5,01	2,72E-03	-	-	-	UP_du_DOWN_dut	-5,28	1,93E-03
flhA	UP_du_DOWN_wt	-8,30	2,30E-05	-	-	-	UP_du_DOWN_dut	-6,78	1,41E-04
flhD	UP_du_DOWN_wt	-5,13	2,79E-03	-	-	-	UP_du_DOWN_dut	-5,14	2,74E-03
flhE	UP_du_DOWN_wt	-5,57	1,65E-03	-	-	-	UP_du_DOWN_dut	-7,05	4,11E-04
ecnB	DOWN_du_UP_wt	4,66	3,79E-03	-	-	-	DOWN_du_UP_dut	3,83	1,41E-02
dctR	DOWN_du_UP_wt	6,56	4,84E-04	-	-	-	DOWN_du_UP_dut	6,25	8,12E-04
otsA	DOWN_du_UP_wt	2,91	4,77E-02	-	-	-	DOWN_du_UP_dut	3,61	1,66E-02
cheW	UP_du_DOWN_wt	-7,71	3,62E-05	-	-	-	UP_du_DOWN_dut	-8,67	1,21E-05
cheR	UP_du_DOWN_wt	-7,35	8,16E-05	-	-	-	UP_du_DOWN_dut	-12,03	5,71E-06
aidB	DOWN_du_UP_wt	5,51	1,30E-03	-	-	-	DOWN_du_UP_dut	5,92	6,49E-04
cheZ	UP_du_DOWN_wt	-8,10	3,20E-05	-	-	-	UP_du_DOWN_dut	-6,01	4,40E-04
glsA	DOWN_du_UP_wt	6,46	4,10E-04	-	-	-	DOWN_du_UP_dut	6,98	1,68E-04
cheY	UP_du_DOWN_wt	-9,14	1,17E-05	-	-	-	UP_du_DOWN_dut	-7,27	7,63E-05
ybaY	-	-	-	-	-	-	DOWN_du_UP_dut	2,91	4,78E-02
cheB	UP_du_DOWN_wt	-11,92	6,93E-06	-	-	-	UP_du_DOWN_dut	-8,73	2,39E-05
cbpA	DOWN_du_UP_wt	3,90	1,22E-02	-	-	-	DOWN_du_UP_dut	4,57	4,17E-03
cheA	UP_du_DOWN_wt	-9,06	4,34E-06	-	-	-	UP_du_DOWN_dut	-8,10	1,48E-05
yodD	DOWN_du_UP_wt	4,64	5,09E-03	-	-	-	DOWN_du_UP_dut	4,79	3,95E-03
cbpM	-	-	-	-	-	-	DOWN_du_UP_dut	3,36	4,53E-02
ybaT	DOWN_du_UP_wt	4,15	9,30E-03	-	-	-	DOWN_du_UP_dut	4,79	3,28E-03
yccJ	DOWN_du_UP_wt	3,65	1,61E-02	-	-	-	DOWN_du_UP_dut	3,83	1,29E-02
ggt	DOWN_du_UP_wt	3,48	3,52E-02	-	-	-	DOWN_du_UP_dut	3,71	2,48E-02
sbp	-	-	-	UP_wt_DOWN_dut	-3,30	3,11E-02	-	-	-
uxuA	UP_du_DOWN_wt	-7,73	2,01E-05	DOWN_wt_UP_dut	7,48	3,20E-05	-	-	-
uxuB	UP_du_DOWN_wt	-8,00	1,37E-05	DOWN_wt_UP_dut	7,96	1,52E-05	-	-	-
cysJ	-	-	-	UP_wt_DOWN_dut	-3,43	2,31E-02	-	-	-
cysD	-	-	-	UP_wt_DOWN_dut	-3,64	1,79E-02	-	-	-
hdeD	DOWN_du_UP_wt	9,07	6,61E-06	-	-	-	DOWN_du_UP_dut	8,86	9,48E-06
hdeB	DOWN_du_UP_wt	7,65	2,80E-05	-	-	-	DOWN_du_UP_dut	7,93	1,73E-05
hdeA	DOWN_du_UP_wt	7,51	3,01E-05	-	-	-	DOWN_du_UP_dut	7,74	1,99E-05
cysU	DOWN_du_UP_wt	3,48	2,50E-02	UP_wt_DOWN_dut	-4,24	8,79E-03	-	-	-
uspB	DOWN_du_UP_wt	3,21	4,16E-02	-	-	-	DOWN_du_UP_dut	3,28	3,77E-02
yehY	DOWN_du_UP_wt	5,13	1,00E-02	-	-	-	DOWN_du_UP_dut	4,97	1,27E-02
cysQ	-	-	-	-	-	-	DOWN_du_UP_dut	2,97	4,87E-02
yhbX	-	-	-	-	-	-	UP_du_DOWN_dut	-4,04	1,57E-02
ves	UP_du_DOWN_wt	-4,71	6,67E-03	-	-	-	UP_du_DOWN_dut	-4,01	1,46E-02
ymfL	DOWN_du_UP_wt	6,63	3,99E-02	-	-	-	-	-	-
flxA	UP_du_DOWN_wt	-4,83	3,09E-03	-	-	-	UP_du_DOWN_dut	-5,42	1,40E-03
iraM	-	-	-	-	-	-	UP_du_DOWN_dut	-6,69	3,40E-02
msyB	DOWN_du_UP_wt	3,16	3,43E-02	-	-	-	-	-	-
argH	-	-	-	UP_wt_DOWN_dut	-3,09	3,92E-02	UP_du_DOWN_dut	-4,74	2,86E-03
argI	-	-	-	UP_wt_DOWN_dut	-3,59	2,70E-02	UP_du_DOWN_dut	-5,55	1,12E-03
valY	-	-	-	-	-	-	UP_du_DOWN_dut	-3,83	3,37E-02
carB	-	-	-	UP_wt_DOWN_dut	-4,55	4,21E-03	UP_du_DOWN_dut	-6,13	2,90E-04

yjiC	UP_du_DOWN_wt	-8,02	4,90E-03	-	-	-	-	-	-
argB	-	-	-	-	-	-	UP_du_DOWN_dut	-4,31	6,18E-03
argC	-	-	-	-	-	-	UP_du_DOWN_dut	-5,03	1,92E-03
argD	-	-	-	-	-	-	UP_du_DOWN_dut	-2,93	4,86E-02
argF	-	-	-	UP_wt_DOWN_dut	-3,61	2,48E-02	UP_du_DOWN_dut	-6,28	3,08E-04
argG	-	-	-	-	-	-	UP_du_DOWN_dut	-4,15	6,97E-03
appY	-	-	-	-	-	-	DOWN_du_UP_dut	3,50	2,56E-02
ydaM	-	-	-	-	-	-	DOWN_du_UP_dut	3,05	4,66E-02
napF	-	-	-	-	-	-	UP_du_DOWN_dut	-4,33	1,68E-02
artJ	-	-	-	-	-	-	UP_du_DOWN_dut	-4,99	1,88E-03
cysW	-	-	-	UP_wt_DOWN_dut	-3,18	3,51E-02	-	-	-
intE	DOWN_du_UP_wt	7,04	2,22E-02	-	-	-	DOWN_du_UP_dut	6,84	2,93E-02
intG	UP_du_DOWN_wt	-7,49	1,96E-04	-	-	-	UP_du_DOWN_dut	-6,58	4,29E-04
ybgS	-	-	-	-	-	-	DOWN_du_UP_dut	3,60	2,07E-02
ygaM	-	-	-	-	-	-	DOWN_du_UP_dut	3,15	3,34E-02
patA	DOWN_du_UP_wt	3,88	1,37E-02	-	-	-	DOWN_du_UP_dut	3,56	2,21E-02
intK	UP_du_DOWN_wt	-3,57	4,84E-02	-	-	-	UP_du_DOWN_dut	-4,49	2,42E-02
cohE	DOWN_du_UP_wt	4,61	1,15E-02	-	-	-	DOWN_du_UP_dut	4,13	2,32E-02
ybgA	DOWN_du_UP_wt	3,42	3,57E-02	-	-	-	DOWN_du_UP_dut	4,23	1,03E-02
yjfY	DOWN_du_UP_wt	3,67	4,84E-02	-	-	-	-	-	-
yegP	DOWN_du_UP_wt	3,14	4,40E-02	-	-	-	DOWN_du_UP_dut	3,42	2,90E-02
fbaB	-	-	-	-	-	-	DOWN_du_UP_dut	3,18	3,21E-02
yceK	DOWN_du_UP_wt	3,93	1,41E-02	-	-	-	DOWN_du_UP_dut	3,38	3,27E-02
yedP	-	-	-	-	-	-	DOWN_du_UP_dut	3,50	2,47E-02
ynfD	-	-	-	-	-	-	DOWN_du_UP_dut	3,83	2,72E-02
mcbR	DOWN_du_UP_wt	5,67	4,40E-03	-	-	-	DOWN_du_UP_dut	5,60	4,79E-03
yedN	UP_du_DOWN_wt	-9,40	5,08E-04	-	-	-	UP_du_DOWN_dut	-4,74	6,32E-03
icdC	UP_du_DOWN_wt	-12,04	5,55E-06	-	-	-	UP_du_DOWN_dut	-12,05	5,50E-06
mdtF	DOWN_du_UP_wt	3,30	2,70E-02	-	-	-	DOWN_du_UP_dut	3,69	1,48E-02
fliZ	UP_du_DOWN_wt	-7,70	3,69E-05	-	-	-	UP_du_DOWN_dut	-7,39	5,46E-05
fliT	UP_du_DOWN_wt	-11,87	7,49E-06	-	-	-	UP_du_DOWN_dut	-6,50	2,51E-04
treB	-	-	-	-	-	-	UP_du_DOWN_dut	-3,45	4,65E-02
fliQ	UP_du_DOWN_wt	-6,08	2,03E-03	-	-	-	UP_du_DOWN_dut	-6,09	2,03E-03
fliP	UP_du_DOWN_wt	-6,85	2,71E-04	-	-	-	UP_du_DOWN_dut	-10,97	3,50E-05
fliS	UP_du_DOWN_wt	-12,03	5,74E-06	-	-	-	UP_du_DOWN_dut	-6,96	1,30E-04
fliR	UP_du_DOWN_wt	-10,06	1,67E-04	-	-	-	UP_du_DOWN_dut	-10,07	1,64E-04
fliM	UP_du_DOWN_wt	-7,61	4,29E-05	-	-	-	UP_du_DOWN_dut	-9,49	6,43E-06
fliL	UP_du_DOWN_wt	-7,46	9,64E-05	-	-	-	UP_du_DOWN_dut	-6,51	2,80E-04
fliO	UP_du_DOWN_wt	-10,46	8,39E-05	-	-	-	UP_du_DOWN_dut	-10,46	8,39E-05
fliN	UP_du_DOWN_wt	-11,37	1,75E-05	-	-	-	UP_du_DOWN_dut	-11,38	1,74E-05
fliI	UP_du_DOWN_wt	-12,05	5,47E-06	-	-	-	UP_du_DOWN_dut	-8,87	1,89E-05
fliH	UP_du_DOWN_wt	-8,77	2,21E-05	-	-	-	UP_du_DOWN_dut	-8,78	2,19E-05
fliK	UP_du_DOWN_wt	-8,49	3,57E-05	-	-	-	UP_du_DOWN_dut	-6,31	3,44E-04
fliJ	UP_du_DOWN_wt	-7,25	2,92E-04	-	-	-	UP_du_DOWN_dut	-7,26	2,92E-04
fliE	UP_du_DOWN_wt	-8,49	3,57E-05	-	-	-	UP_du_DOWN_dut	-7,03	1,44E-04
fliD	UP_du_DOWN_wt	-7,74	2,38E-05	-	-	-	UP_du_DOWN_dut	-8,73	6,29E-06
fliG	UP_du_DOWN_wt	-9,11	5,72E-06	-	-	-	UP_du_DOWN_dut	-10,03	2,53E-06
fliF	UP_du_DOWN_wt	-13,53	4,27E-07	-	-	-	UP_du_DOWN_dut	-10,35	1,48E-06
fliA	UP_du_DOWN_wt	-9,10	3,28E-06	-	-	-	UP_du_DOWN_dut	-10,07	1,09E-06
fliC	UP_du_DOWN_wt	-8,14	8,27E-06	-	-	-	UP_du_DOWN_dut	-8,05	9,80E-06
crfC	UP_du_DOWN_wt	-5,00	2,01E-03	-	-	-	UP_du_DOWN_dut	-3,59	1,78E-02
gntP	UP_du_DOWN_wt	-6,31	4,76E-04	DOWN_wt_UP_dut	4,41	1,08E-02	-	-	-
yahO	DOWN_du_UP_wt	3,93	1,41E-02	-	-	-	DOWN_du_UP_dut	4,24	8,65E-03

slp	DOWN_du_UP_wt	6,59	2,11E-04	-	-	-	DOWN_du_UP_dut	6,68	1,80E-04
yahK	DOWN_du_UP_wt	3,49	2,47E-02	-	-	-	DOWN_du_UP_dut	3,46	2,53E-02
ydfK	DOWN_du_UP_wt	5,21	8,95E-03	-	-	-	DOWN_du_UP_dut	5,27	8,04E-03
yiaG	DOWN_du_UP_wt	3,23	3,48E-02	-	-	-	DOWN_du_UP_dut	3,37	2,79E-02
csgC	UP_du_DOWN_wt	-6,57	3,99E-02	-	-	-	-	-	-
tap	UP_du_DOWN_wt	-10,28	1,64E-06	-	-	-	UP_du_DOWN_dut	-8,41	1,09E-05
tar	UP_du_DOWN_wt	-8,97	5,06E-06	-	-	-	UP_du_DOWN_dut	-9,54	2,75E-06
cbdB	DOWN_du_UP_wt	4,11	1,82E-02	-	-	-	-	-	-
cbdA	DOWN_du_UP_wt	4,27	1,44E-02	-	-	-	-	-	-
modC	UP_du_DOWN_wt	-3,31	2,91E-02	-	-	-	-	-	-
arrS	DOWN_du_UP_wt	6,21	1,81E-03	-	-	-	DOWN_du_UP_dut	6,46	1,18E-03
sdhC	-	-	-	-	-	-	UP_du_DOWN_dut	-3,39	3,46E-02
ygaU	DOWN_du_UP_wt	3,25	2,97E-02	-	-	-	DOWN_du_UP_dut	3,94	1,11E-02
purL	-	-	-	UP_wt_DOWN_dut	-3,65	1,75E-02	UP_du_DOWN_dut	-3,81	1,31E-02
ytjA	DOWN_du_UP_wt	4,30	6,62E-03	-	-	-	DOWN_du_UP_dut	3,92	1,19E-02
motA	UP_du_DOWN_wt	-9,95	2,89E-06	-	-	-	UP_du_DOWN_dut	-9,04	6,42E-06
motB	UP_du_DOWN_wt	-7,87	2,75E-05	-	-	-	UP_du_DOWN_dut	-12,94	1,18E-06
adhP	-	-	-	-	-	-	DOWN_du_UP_dut	3,43	2,48E-02
yagI	-	-	-	-	-	-	UP_du_DOWN_dut	-3,77	1,77E-02
yohC	DOWN_du_UP_wt	5,59	5,00E-03	-	-	-	DOWN_du_UP_dut	6,14	2,03E-03
yeeE	-	-	-	UP_wt_DOWN_dut	-4,53	4,74E-03	-	-	-
clsB	DOWN_du_UP_wt	3,74	3,16E-02	-	-	-	DOWN_du_UP_dut	4,47	1,05E-02
yohF	DOWN_du_UP_wt	3,26	4,84E-02	-	-	-	DOWN_du_UP_dut	3,69	2,56E-02
lit	DOWN_du_UP_wt	8,28	3,11E-03	-	-	-	DOWN_du_UP_dut	7,94	5,67E-03
ygiW	DOWN_du_UP_wt	6,30	3,79E-04	-	-	-	DOWN_du_UP_dut	6,37	3,34E-04
ymgE	-	-	-	-	-	-	DOWN_du_UP_dut	4,52	2,63E-02
curA	-	-	-	-	-	-	DOWN_du_UP_dut	3,04	4,74E-02
yjdI	DOWN_du_UP_wt	7,13	1,96E-02	-	-	-	DOWN_du_UP_dut	7,28	1,55E-02
ddpF	-	-	-	-	-	-	DOWN_du_UP_dut	3,47	2,98E-02
ycgB	DOWN_du_UP_wt	3,26	3,23E-02	-	-	-	DOWN_du_UP_dut	3,96	1,10E-02
yjdN	-	-	-	-	-	-	DOWN_du_UP_dut	3,62	3,16E-02
sdaB	-	-	-	-	-	-	UP_du_DOWN_dut	-2,91	4,85E-02
sdaC	-	-	-	-	-	-	UP_du_DOWN_dut	-3,28	2,82E-02
mglA	-	-	-	-	-	-	UP_du_DOWN_dut	-6,45	4,72E-02
gabT	-	-	-	-	-	-	DOWN_du_UP_dut	3,70	2,34E-02
ykgF	DOWN_du_UP_wt	6,74	3,40E-02	-	-	-	DOWN_du_UP_dut	6,84	2,93E-02
ybhP	DOWN_du_UP_wt	4,46	1,45E-02	-	-	-	DOWN_du_UP_dut	4,91	7,15E-03
ymfI	DOWN_du_UP_wt	4,28	3,77E-02	-	-	-	DOWN_du_UP_dut	4,63	2,23E-02
ymfJ	DOWN_du_UP_wt	7,04	2,22E-02	-	-	-	-	-	-
talA	DOWN_du_UP_wt	3,46	2,14E-02	-	-	-	DOWN_du_UP_dut	4,14	7,41E-03
otsB	DOWN_du_UP_wt	4,15	8,76E-03	-	-	-	DOWN_du_UP_dut	4,75	3,29E-03
pyrI	-	-	-	-	-	-	UP_du_DOWN_dut	-3,84	1,22E-02
ydhS	-	-	-	-	-	-	DOWN_du_UP_dut	2,99	4,95E-02
phr	-	-	-	-	-	-	DOWN_du_UP_dut	3,08	4,20E-02
gabD	-	-	-	-	-	-	DOWN_du_UP_dut	3,27	3,96E-02
treF	-	-	-	-	-	-	DOWN_du_UP_dut	3,10	4,11E-02
ymfD	DOWN_du_UP_wt	6,50	4,72E-02	-	-	-	-	-	-
pyrB	-	-	-	-	-	-	UP_du_DOWN_dut	-4,34	5,45E-03
rhsA	DOWN_du_UP_wt	4,14	4,61E-02	-	-	-	-	-	-
yhiD	DOWN_du_UP_wt	6,11	4,75E-04	-	-	-	DOWN_du_UP_dut	6,20	4,08E-04
carA	-	-	-	UP_wt_DOWN_dut	-3,39	3,29E-02	UP_du_DOWN_dut	-5,18	1,85E-03
yhiM	DOWN_du_UP_wt	10,21	1,28E-04	-	-	-	DOWN_du_UP_dut	10,91	3,87E-05
ycfQ	-	-	-	-	-	-	UP_du_DOWN_dut	-3,23	3,44E-02

flgC	UP_du_DOWN_wt	-13,18	7,86E-07	-	-	-	UP_du_DOWN_dut	-13,18	7,81E-07
flgB	UP_du_DOWN_wt	-8,97	7,20E-06	-	-	-	UP_du_DOWN_dut	-13,09	9,12E-07
flgA	UP_du_DOWN_wt	-7,35	5,82E-05	-	-	-	UP_du_DOWN_dut	-8,63	1,31E-05
ybhN	DOWN_du_UP_wt	4,14	4,61E-02	-	-	-	DOWN_du_UP_dut	4,69	2,07E-02
flgG	UP_du_DOWN_wt	-8,55	7,41E-06	-	-	-	UP_du_DOWN_dut	-9,27	3,02E-06
flgF	UP_du_DOWN_wt	-9,59	2,50E-06	-	-	-	UP_du_DOWN_dut	-9,60	2,48E-06
flgE	UP_du_DOWN_wt	-9,44	1,34E-06	-	-	-	UP_du_DOWN_dut	-9,67	9,82E-07
flgD	UP_du_DOWN_wt	-9,59	1,74E-06	-	-	-	UP_du_DOWN_dut	-14,26	1,20E-07
flgK	UP_du_DOWN_wt	-9,33	2,71E-06	-	-	-	UP_du_DOWN_dut	-9,90	1,47E-06
flgJ	UP_du_DOWN_wt	-7,12	8,66E-05	-	-	-	UP_du_DOWN_dut	-7,44	5,76E-05
flgI	UP_du_DOWN_wt	-7,71	3,60E-05	-	-	-	UP_du_DOWN_dut	-8,68	1,21E-05
flgH	UP_du_DOWN_wt	-8,72	1,11E-05	-	-	-	UP_du_DOWN_dut	-7,77	3,26E-05
flgN	UP_du_DOWN_wt	-5,71	6,42E-04	-	-	-	UP_du_DOWN_dut	-4,97	2,00E-03
flgM	UP_du_DOWN_wt	-4,91	2,45E-03	-	-	-	UP_du_DOWN_dut	-5,03	2,05E-03
flgL	UP_du_DOWN_wt	-7,19	5,31E-05	-	-	-	UP_du_DOWN_dut	-6,33	2,03E-04
