# Supplementary material for: Sense and antisense RNA products of the uxuR gene can affect motility and chemotaxis acting independent of the UxuR protein
Source: Front Mol Biosci. 2023 Feb 17;10:1121376. doi: 10.3389/fmolb.2023.1121376 (PMC10016265; doi:10.3389/fmolb.2023.1121376)
Supplement: Supplementary file 2 [file DataSheet1.pdf]

Supplementary Material

Supplemetary Table S1. Primers used for amplification of regions containing predicted intragenic promoters and the genomic context of the respective genes. Genes where promoters for antisense or co-directed transcripts were predicted, and their direction are in bold. Restriction sites for BglII and XbaI in the hnsP primers are underlined.

Primer (strand)	Sequence	Potential RNA(s) strand	Gene genomic context
dps (-)	5' – TGGGCGGTGTAGCTCTGGGG – 3'	+	←*glnH*←***dps***←*rhtA*
dps_new (-)	5'-CGATCATCTGGATACCATGG-3'		
dps_s (+)	5' – AAGTGCGTTGAGGTGGGCTG – 3'		
dps_s_new (+)	5'-TATTCGATGTTAGACTCGAT-3'		
hns (-)	5' – GAAGTTGAAGAGCGCACTCG – 3'	+	*galU*→←***hns***→(650bp) *tdk*
hns_new (-)	5'-TCTGAACAACATCCGTACTCTTCG-3'		
hns_s (+)	5' – ACAGCTGGAGTACGGCCTTG – 3'		
hns_s_new (+)	5'-TACATGCAGGCCTTCGTTGA-3'		
hnsP_F (-)	5'-ATTTAGATCTCCTTACATTCCTG-3'	-, gene promoter	*galU*→←***hns***→(650bp) *tdk*
hnsP_R (+)	5'-GTTGTCTAGAATTTTAAGTGCTTCG-3'		
lacZ (-)	5'-CAACCCGTGGTCGGCTTACG-3'	+	←*lacY*←***lacZ***←*lacI*
lacZ_s (+)	5'-CCATCCAGTGCAGGAGCTCG-3'		
phoR (+)	5'-GCGCGTTCCCGGCAAACCGG-3'	-	*phoR*→***phoR***→*brnQ*→
phoR_new (+)	5'-CGGTAGCGGATTAGGGTTAG-3'		
phoR_s (-)	5'-CTGTAAGCCGACCATTGGAG-3'		
phoU_new (-)	5'-CTACTACGTGAAGGGGCAGG-3'	+	←*bglG*←***phoU***←*pstB*
phoU (-)	5'-CGAGCGTACTTACTGCGCTG-3'		
phoU_s (+)	5'-GCGCGTTTTTGAAAGCCAATTC-3'		
phoU_s_new (+)	5'-GAACATCCAGGTTCGGAAATTA-3'		
rcsA (+)	5'-TCTCTCGACGATATCCTTGG-3'	-	*fliR*→***rcsA***→←*dsrB*
rcsA_s (-)	5'-TCAGTCGGACGACATGGTAG-3'		

rho (+)	5'-CATGGAACTGCACCTCTCTC-3'	-	*rhoL*→***rho***→*rfe*→
rho_new (+)	5'-CTTCCCGGCTATCGACTACA-3'		
rho_s (-)	5'-CAGAAGTATAACCACGAAGAC-3'		
uxuR (-)	5' – TATCGCAAAGAGTGGTTGGG – 3'	-.+	*uxuAB*→***uxuR***→←*yjiC*
uxuR_new (+)	5'-GAGCTGTTCCGTCAGTCCTG-3'		
uxuR_s (-)	5'-CCAGGAAGAATGAGTACTAAC-3'		
uxuR_s_new (-)	5'-GAAAATGCACCACTCAAACG-3'		
uxuR_P (-)	5'-AGGATTGTACGGCGTCTTTATGA-3'	+, gene promoter	*uxuAB*→***uxuR***→←*yjiC*
uxuR_P_new (-)	5' –CATGATCAGCGCTTCACGTA-3'		
uxuR_P_s-43 (+)	5'-ACCTGTACGTGCCAACTTCCA-3'		
uxuR_P_s-130 (+)	5'-TGGCTGCGCTGTAACTAACTG-3'		
arcA-F1 (-)	5'-GAGTTGGTAACACGCAACACG-3'	-	*creD*→←***arcA***→*yjjY*
arcA-R1 (+)	5'-CAGTTCACGCGGGTTGAACGG-3'		
birA-F1 (+)	5'-GGCGGCCATGCTAATACGTG-3'	+	*murB*→***birA***→←*coaA*
birA-R1(-)	5'-CGGTAGAAGAGGTCAGACTACGC-3'		
fucR-F1 (+)	5'-GGCTGTTACGTCAATCCCTCGC-3'	+	*fucU*→***fucR***→←*rlmM*
fucR-F2 (+)	5'-GAAGGGATCGATAGCAGCGG-3'		
fucR-R1 (-)	5'-CGTCTCCGGCCTGCTACCCT-3'		
htgA-F (+)	5'-CACCCTGGCATCGACCGCGT-3'	+	←*satP*←*yaaW* (***htgA***→)←*yaaI*
htgA-R (-)	5'-GGGCATCCCGAGCAACATCG-3'		
tyrR-F (+)	5'-TCGCCTGCGCAACCATACCG-3'	+	*ycjF*→***tyrR***→←*tpx*
tyrR-R2 (-)	5'-AGGCGCTGACGTCCTGGGCG-3'		
tyrR-R4 (-)	5'-CAATTGCTGATGGCCCACTGA-3'		

Supplemetary Table S2. Primers used for amplification of the *uxuR* regions containing predicted antisense and co-directed promoters

Primer	Sequence
1 (uxuR_antis)	5’-AGCATCTGGAAAACGTTAAG-3’
2 (uxuR)	5’ – TATCGCAAAGAGTGGTTGGG – 3’
3 (uxuR_new)	5’-GAGCTGTTCCGTCAGTCCTG-3’
4 (uxuR_codir)	5’ – ATTTACAAAGTAATACTCATTG-3’
5 (uxuR_s)	5’-CCAGGAAGAATGAGTACTAAC-3’
6 (uxuR_s_new)	5’-GAAAATGCACCACTCAAACG-3’

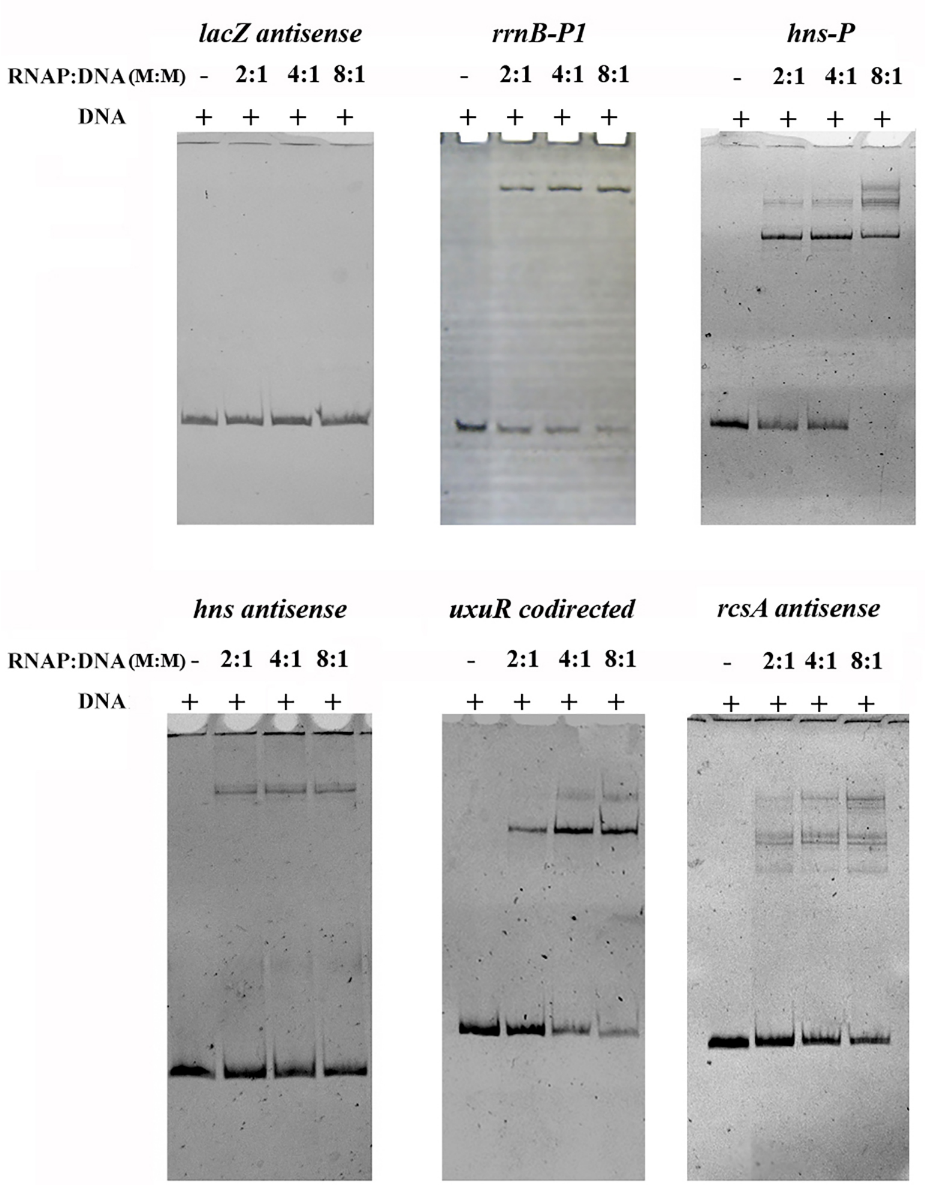

Supplementary Figure 1. An example of the band-shift experiments with RNA polymerase and DNA fragments containing known promoters (*rrnB*-P1 and *hns*-P), and predicted promoters for synthesis of antisense (in the *dps* and *rcsA* genes, *lacZ* is shown as an example of a negative result) and co-directed (in *uxuR*) RNAs. Molar ratios are shown above the lanes.

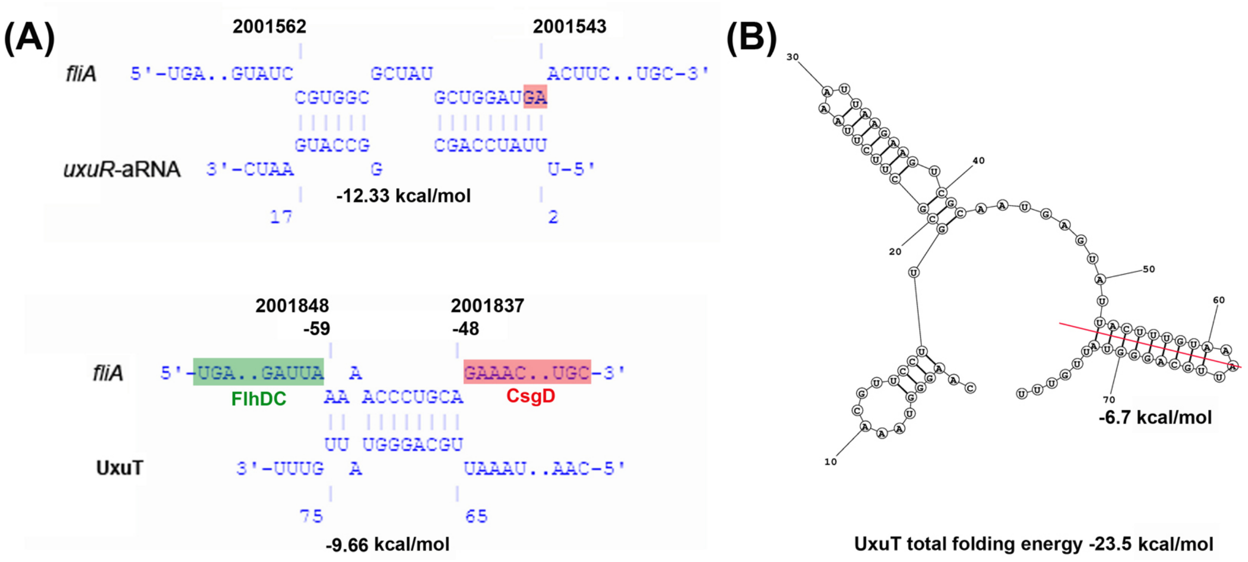

Supplementary Figure 2 (A) Predicted binding of *uxuR*-aRNA and UxuT to the *fliA* mRNA and promoter region. In the lower structure, green indicates activator function of the respective protein, while red indicates repression. **(B)** Predicted structure of UxuT. Part of the stem-loop hybridising with *fliA* is divided by the red line.
